# Molecular and cognitive signatures of ageing partially restored through synthetic delivery of IL2 to the brain

**DOI:** 10.15252/emmm.202216805

**Published:** 2023-03-28

**Authors:** Pierre Lemaitre, Samar HK Tareen, Emanuela Pasciuto, Loriana Mascali, Araks Martirosyan, Zsuzsanna Callaerts‐Vegh, Suresh Poovathingal, James Dooley, Matthew G Holt, Lidia Yshii, Adrian Liston

**Affiliations:** ^1^ VIB Center for Brain and Disease Research Leuven Belgium; ^2^ Department of Microbiology, Immunology and Transplantation KU Leuven Leuven Belgium; ^3^ Immunology Programme The Babraham Institute Babraham UK; ^4^ Department of Neurosciences KU Leuven Leuven Belgium; ^5^ Laboratory of Biological Psychology, Faculty of Psychology KU Leuven Leuven Belgium; ^6^ Department of Pathology The University of Cambridge Cambridge UK; ^7^ Instituto de Investigaçāo e Inovaçāo em Saúde (i3S) University of Porto Porto Portugal

**Keywords:** Aging, Brain, Gene Therapy, Interleukin 2, Regulatory T cells, Immunology, Neuroscience

## Abstract

Cognitive decline is a common pathological outcome during aging, with an ill‐defined molecular and cellular basis. In recent years, the concept of inflammaging, defined as a low‐grade inflammation increasing with age, has emerged. Infiltrating T cells accumulate in the brain with age and may contribute to the amplification of inflammatory cascades and disruptions to the neurogenic niche observed with age. Recently, a small resident population of regulatory T cells has been identified in the brain, and the capacity of IL2‐mediated expansion of this population to counter neuroinflammatory disease has been demonstrated. Here, we test a brain‐specific IL2 delivery system for the prevention of neurological decline in aging mice. We identify the molecular hallmarks of aging in the brain glial compartments and identify partial restoration of this signature through IL2 treatment. At a behavioral level, brain IL2 delivery prevented the age‐induced defect in spatial learning, without improving the general decline in motor skill or arousal. These results identify immune modulation as a potential path to preserving cognitive function for healthy aging.

## Graphical abstract

Delivery of interleukin 2 into the brain via a blood–brain barrier crossing AAV vector prevents cognitive decline in the aging mice.

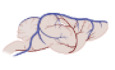

**The paper explained**

**The problem**

Declining cognitive health is near‐ubiquitous in the aged, with normal decline accelerated to mild cognitive impairment in ~ 40% of individuals over the age of 60. The biological causes of cognitive decline in aging are complex and incompletely understood. However, there is a growing appreciation that chronic low‐grade inflammation is a key driver. One potential counter to this inflammatory process is to leverage the anti‐inflammatory properties of the protein interleukin 2. Delivery of interleukin 2 to the brain, using a gene delivery system, expands the endogenous brain‐resident population of regulatory T cells. This treatment has proven efficacious in mouse models of traumatic brain injury, stroke, and multiple sclerosis, but whether it would also counter normal cognitive decline during aging was unknown.

**Results**

We treated aged mice with interleukin 2, using an AAV‐based viral vector to give brain‐specific gene expression. Aging of the brain was assessed through molecular profiling of the glial compartment of the brain—microglia, astrocytes, and oligodendrocytes, and through testing of cognitive performance. Treatment of aged mice with interleukin 2 partially reversed the aging signature in glial cells, restoring key pathways to the state observed in young mice. At a cognitive level, treatment prevented the age‐induced decline in spatial learning characteristic of wild‐type mice, with treated aged animals performing nearly as well as young animals in challenge tests.

**Impact**

Mild cognitive decline is a growing problem of global impact. For the ~ 40% of individuals over the age of 60 with mild cognitive decline, the loss of cognitive function can be frustrating and anxiety‐inducing; a third of those individuals progress to dementia within 5 years, losing the capacity for independent living. Gene delivery systems have the potential to give long‐term sustained local delivery of anti‐inflammatory proteins to the brain. These results provide proof‐of‐principle for using gene delivery of IL2 and expansion of regulatory T cells to halt mild cognitive decline in aged individuals.

## Introduction

Aging is an irreversible process associated with physical deterioration and hence especially noticeable in organs containing mainly postmitotic cells, such as the brain. The progressive decline in memory, orientation, attention, motivation, and cognition seen in humans is thought to stem largely from aging‐related processes (Hof & Morrison, 2004). Although cognitive aging research generally focuses on neurons, which are widely held to be the computational units of the brain, there is an increasing realization that reciprocal interactions between neurons and non‐neuronal cells (collectively referred to as glia) play key roles in all aspects of nervous system function. These include the regulation of synapse formation and function by astrocytes, as well as myelination and provision of trophic support to neurons by oligodendrocytes. Once thought of as solely immune cells, which protect the CNS against injury and disease, there is increasing evidence that the same systems used by microglia to remove invading pathogens from the brain have also been co‐opted to remove weak and/or inappropriate neuronal synapses, establishing precise patterns of neuronal connectivity in the brain (Allen & Lyons, 2018). It should not be surprising, therefore, that aging‐related cognitive decline has been associated with several molecular processes, including chronic inflammation, impaired autophagy, macromolecular damage, and mitochondrial dysfunction and senescence (Hou *et al*, 2019), which impact the function of all CNS cell types (Jurk *et al*, 2012; Bussian *et al*, 2018; Chinta *et al*, 2018; Ogrodnik *et al*, 2019).

In addition to the shared cellular phenotypes of aging, unique phenotypes emerge among the individual glial cell types with aging. Microglia from aged animals are hyper‐reactive to inflammatory stimuli, with higher basal levels of cytokine expression and exaggerated responses to activation (Deczkowska *et al*, 2017). Aged microglia exhibit shortening of their membranous processes, slower process extension and retraction, and enlarged soma volumes (Hefendehl *et al*, 2014). Functional decline of microglia is hypothesized to be progressive over the lifespan, due to the accumulation of oxidative DNA damage and nondegradable protein and lipid aggregates. This inflamed and dysfunctional microglial profile plays a role in the development of age‐associated neurodegenerative diseases (Spittau, 2017). This aging phenotype of microglia is driven in part by the breakdown of myelin (Safaiyan *et al*, 2016), a phenomenon paralleled by the aging of oligodendrocytes. Oligodendrocytes are derived from specific neural progenitor cells, oligodendrocyte progenitor cells (OPCs), with the primary role of myelin production. OPC heterogeneity increases with age (Spitzer *et al*, 2019), while the myelination function of oligodendrocytes is decreased (Schain *et al*, 2014), with an associated breakdown in myelin integrity (Peters *et al*, 1996). Astrocytes undergo substantial morphological and transcriptional changes with aging, consistent with the acquisition of a reactive proinflammatory phenotype (Robillard *et al*, 2016; Clarke *et al*, 2018), although there are regional differences in the response of astrocytes across different areas of the brain (Rodriguez‐Arellano *et al*, 2016). The degree to which these glial changes are drivers, mediators or bystanders in the cellular pathways linking aging to cognitive decline is debated.

One of the most recognized effects of aging is dysregulation of the immune system. Both immunosenescence, defective initiation and resolution of immune responses, and inflammaging occur with age (Montecino‐Rodriguez *et al*, 2013; Deleidi *et al*, 2015). Aging affects both the adaptive and the innate immune system (Shaw *et al*, 2013; Carrasco *et al*, 2021). In the brain, immune aging is accompanied by a low‐grade chronic proinflammatory environment, characterized by increased production of proinflammatory cytokines, such as interleukin‐6 (IL‐6) and tumor necrosis factor alpha (TNFα), acute‐phase proteins, reactive oxygen species (ROS), and autoantibodies. Lymphatic drainage of inflammatory products from the brain becomes insufficient with age, contributing to cognitive decline (Da Mesquita *et al*, 2018). CNS leukocytes may also play a role in age‐related inflammation and neurodegeneration (Baruch *et al*, 2013; Prokop *et al*, 2015; Ritzel *et al*, 2016). In particular, the accumulation of clonally expanded T cells is observed in the aged brain, accompanied by the production of IFNγ (Dulken *et al*, 2019) and loss of CCR7 (Da Mesquita *et al*, 2021). The inflammatory effects of T cells in the brain in turn impede the neuronal stem cell niche and alter the biology of the glial compartment, amplifying the effects of neuroaging (Dulken *et al*, 2019).

Beyond the observed age‐associated cognitive decline, aging is also a major risk factor for pathological neurodegeneration. The risk of neurodegenerative diseases such as Alzheimer's disease (AD), Parkinson's disease (PD), amyotrophic lateral sclerosis (ALS), and primary progressive multiple sclerosis (PPMS) increases significantly in the elderly population (Hou *et al*, 2019). This enhanced rate of neurodegenerative disease is associated with neuroinflammation, characterized by reactive central nervous system microglia and astroglia, as well as infiltrating peripheral monocytes and lymphocytes. The presence of vascular and parenchymal T cells in brains of postmortem AD patients (Merlini *et al*, 2018) and in transgenic models of AD (Ferretti *et al*, 2016) suggests the potential involvement of T cells in the development of neuroinflammation. In PD, T cells have been observed in the substantia nigra of patients, and pharmacological manipulation of T cell responses alters disease outcome in mouse models (Baird *et al*, 2019). Likewise, T cell infiltration is observed at sites of motor neuron loss in ALS patients, with modified disease progression in T cell‐deficient mouse models (Zhao *et al*, 2013). The association of HLA alleles to AD and PD susceptibility supports a causative role of T cells in the process (Hamza *et al*, 2010; International Parkinson Disease Genomics Consortium *et al*, 2011; Lambert *et al*, 2013). Together, this suggests that infiltrating T cells in the brain contribute to both common age‐related cognitive decline and a diversity of neurodegenerative diseases.

Regulatory T cells (Treg) are a subset of T cells with potent anti‐inflammatory and pro‐repair functions. Treg reside in both lymphoid and in nonlymphoid organs, where they exert diverse functions regulating tissue homeostasis and contributing to tissue repair (Liston & Gray, 2014). Tregs are found in low numbers in the mouse and human brain and are observed in both the parenchyma and meningeal lymphatics (Liston *et al*, 2022). Tregs have been proposed to have a protective function in a variety of neuroinflammatory and/or neurodegenerative diseases. Proposed therapeutic strategies to harness Tregs are typically based on either direct delivery of Tregs, through cell‐based approaches, or the provision of biologics to enhance Tregs, often IL2, the key survival factor (Pierson *et al*, 2013). The results have, however, been inconsistent across models and with different modalities of treatment. For example, in AD, direct cell therapy with Tregs (Baek *et al*, 2016; Faridar *et al*, 2022; Yang *et al*, 2022) has given beneficial effects, although other studies have shown the reverse (Baruch *et al*, 2015; Yang *et al*, 2020), while treatment with IL2 has given conflicting results, depending on the AD model, ranging from protective (Baek *et al*, 2016; Alves *et al*, 2017) to minor (Dansokho *et al*, 2016) or no effect (preprint: Yshii *et al*, 2022a). In PD models, a number of different Treg‐based treatments have been beneficial, including direct cell transfer (Reynolds *et al*, 2007; Huang *et al*, 2020; Markovic *et al*, 2022), using bee venom phospholipase A2 to induce Treg formation, superagonist anti‐CD28 antibodies to expand them (Badr *et al*, 2022) or vasoactive intestinal peptide receptor‐2 (VIPR2) peptide agonist to enhance activity (Mosley *et al*, 2019). In stroke models, beneficial effects have been shown with direct Treg cell therapy (Ito *et al*, 2019) (although this only works in Rag‐deficient mice), or via the use of IL33 (Liu *et al*, 2020) or IL2 to expand the Treg population (Zhang *et al*, 2018; Dong *et al*, 2021; Shi *et al*, 2021; Yshii *et al*, 2022b). Finally, in ALS models, both Treg cell therapy (Beers *et al*, 2011) and IL2 treatment (Sheean *et al*, 2018) have shown beneficial results. While supporting a key role for Tregs in preventing pathology in neurodegenerative diseases, many of these studies use systems that do not distinguish between direct neuroprotection, via brain‐resident Tregs, or indirect protection, through control of peripheral inflammatory reaction by systemic Tregs.

Despite the growing recognition of a protective role of IL2 and Tregs in a range of neuroinflammatory and neurodegenerative processes, the pathological processes occurring during aging are distinct from those in disease, and thus the potential of these cells to protect the aging brain remains unknown. Here, we used the synthetic delivery of brain‐specific IL2, which was recently demonstrated to protect against neuroinflammatory injury and disease (Yshii *et al*, 2022b), to test the hypothesis that neurological aging can be restrained by modulation of the immunological compartment of the brain. Using young and aged mice, we characterized the molecular signature of aging in the brain glial compartment and found IL2 capable of restraining a core component of the molecular aging process across glial types. These molecular effects were associated with a partial preservation of cognitive capacity, suggesting that brain‐specific IL2 production may be beneficial in combating age‐related cognitive decline.

## Results

### Age drives a change in the cellular composition of brain‐resident glia

**Figure 1:**
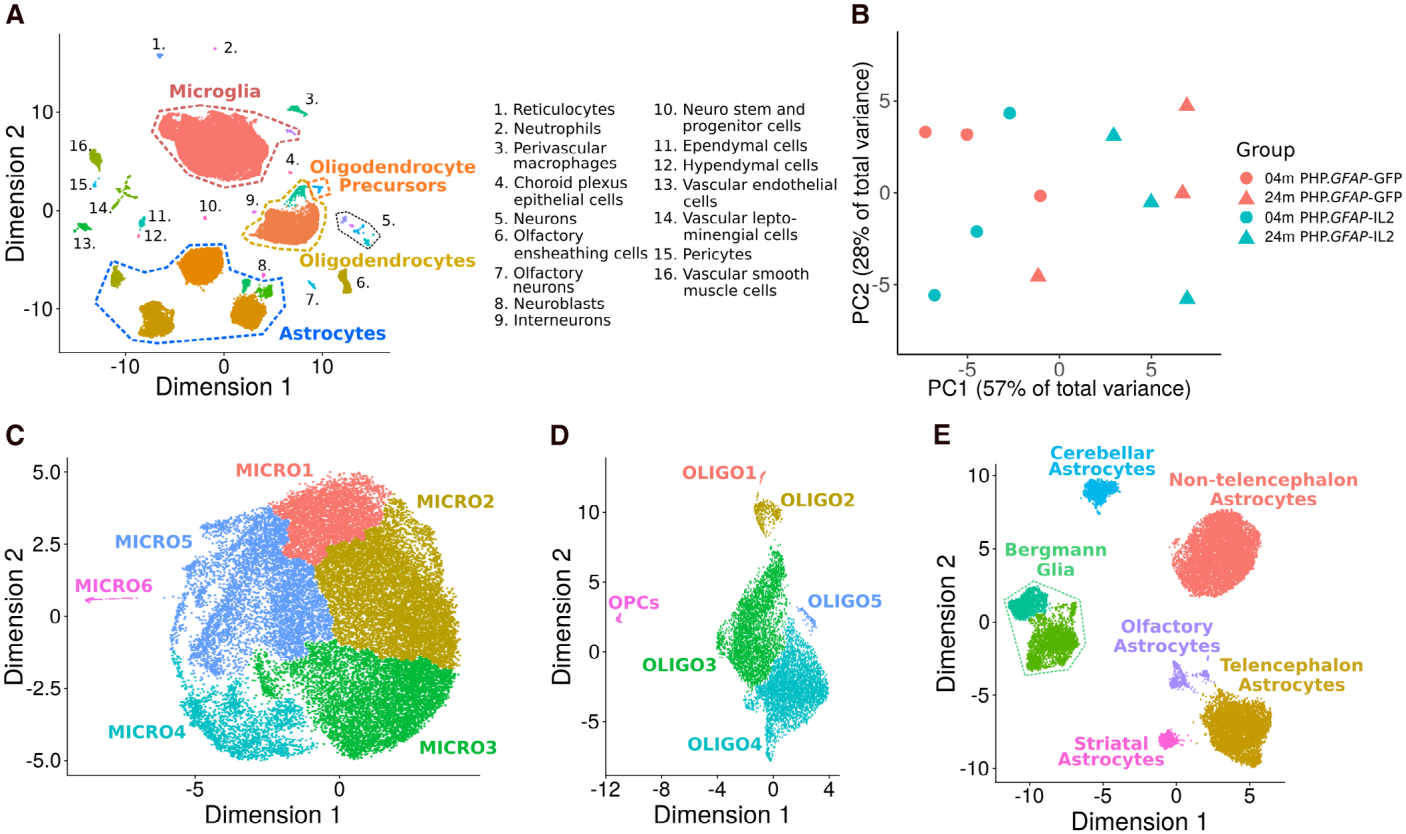
Single‐cell sequencing identifies a shift in glia proportions with age Wild‐type mice were treated with PHP.*GFAP*‐IL2 (or PHP.*GFAP*‐GFP control vector) at 2 months or 22 months of age (*n* = 3/group). Two‐month post‐treatment, the glial compartment was sorted from perfused mice and characterized using 10× single‐cell transcriptomics.
UMAP projection of the cells in the dataset. Colored names indicate the glial populations sorted for downstream analysis (as in Appendix Fig S1), whereas minor populations annotated in black are contaminant cell types found in the data postsorting (as in Appendix Fig S2).PCA was performed on the proportions of cells from each sample in each cluster to generate an overview of whether age and/or treatment show any global trend with respect to sample proportions in each cluster of the data.The microglia clusters were reclustered and projected in UMAP space to generate further separation in the data.The oligodendrocyte and oligodendrocyte precursor (OPC) clusters were reclustered and projected in UMAP space to generate further separation in the data.The astrocyte clusters were reclustered and reprojected in UMAP space. The different astrocyte clusters were then mapped onto known astrocyte subtypes.

**Figure EV1:**
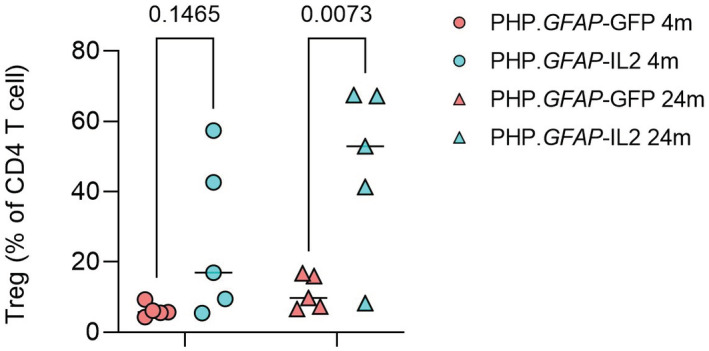
PHP.*GFAP*‐IL2 expands brain‐resident regulatory T cells in aged mice Wild‐type mice were treated with PHP.*GFAP*‐GFP control vector or PHP.*GFAP*‐IL2 at 2 months or 22 months of age, and were assessed for brain‐resident Foxp3 + CD4+ Tregs by flow cytometry 2 months late (4 or 24 months of age). *N* = 5/group. Data information: Mean and individual data points. 2‐way ANOVA repeated measures with age and treatment as the main factors. Source data are available online for this figure.

A key limitation to proposed Treg‐ or IL2‐based therapies is the peripheral impact of most proposed strategies, with the potential for undesirable peripheral immunosuppression. The use of brain‐targeted delivery opens the door to potential use to dampen down the inflammation associated with various neuropathologies, while preserving the integrity of the peripheral immune system. One way to achieve brain‐specific expression of IL2 is through the gene delivery vector PHP.*GFAP*‐IL2. This approach results in the restricted production of IL2 in the brain and an accompanying expansion of brain‐resident Tregs (Yshii *et al*, 2022b). PHP.*GFAP*‐IL2 treatment is protective against neuroinflammation in the context of traumatic brain injury, stroke, and experimental autoimmune encephalitis (Yshii *et al*, 2022b). In order to test the influence of brain‐specific IL2 delivery on aging, we treated young mice, at 2 months of age, and old mice, at 22 months of age, with PHP.*GFAP*‐IL2 or the control PHP.*GFAP*‐GFP vector. As previously demonstrated on young mice (Yshii *et al*, 2022b), this treatment expanded the frequency of Tregs in the brain of aged mice (Fig EV1). The effect of this treatment was assessed on the glial compartment of the whole brain 2 months later, at 4 and 24 months of age, through the flow cytometric sorting and single‐cell RNA sequencing of microglia, astrocytes, and oligodendrocytes (Fig 1A). The target glial populations (Appendix Fig S1) and minor population contaminants (Appendix Fig S2) were identified through expression analysis of key lineage markers and integration with reference datasets (La Manno *et al*, 2021). PCA analysis of major and minor population frequencies found an association with age but not treatment, indicating an age‐dependent phenotype (Fig 1B). Extraction and reclustering of microglia resulted in tightly clustered cells (Fig 1C), while reclustering of oligodendrocytes led to a clear separation of oligodendrocyte precursors (OPCs) and oligodendrocytes (Fig 1D). Extraction and reclustering of astrocytes, by contrast, produced a clear transcriptional separation of Bergmann glia, Cerebellar astrocytes, Olfactory astrocytes, striatal astrocytes, telencephalon astrocytes, and nontelencephalon astrocytes (Fig 1E), based on key markers and dataset integration (Appendix Fig S3).

**Figure 2:**
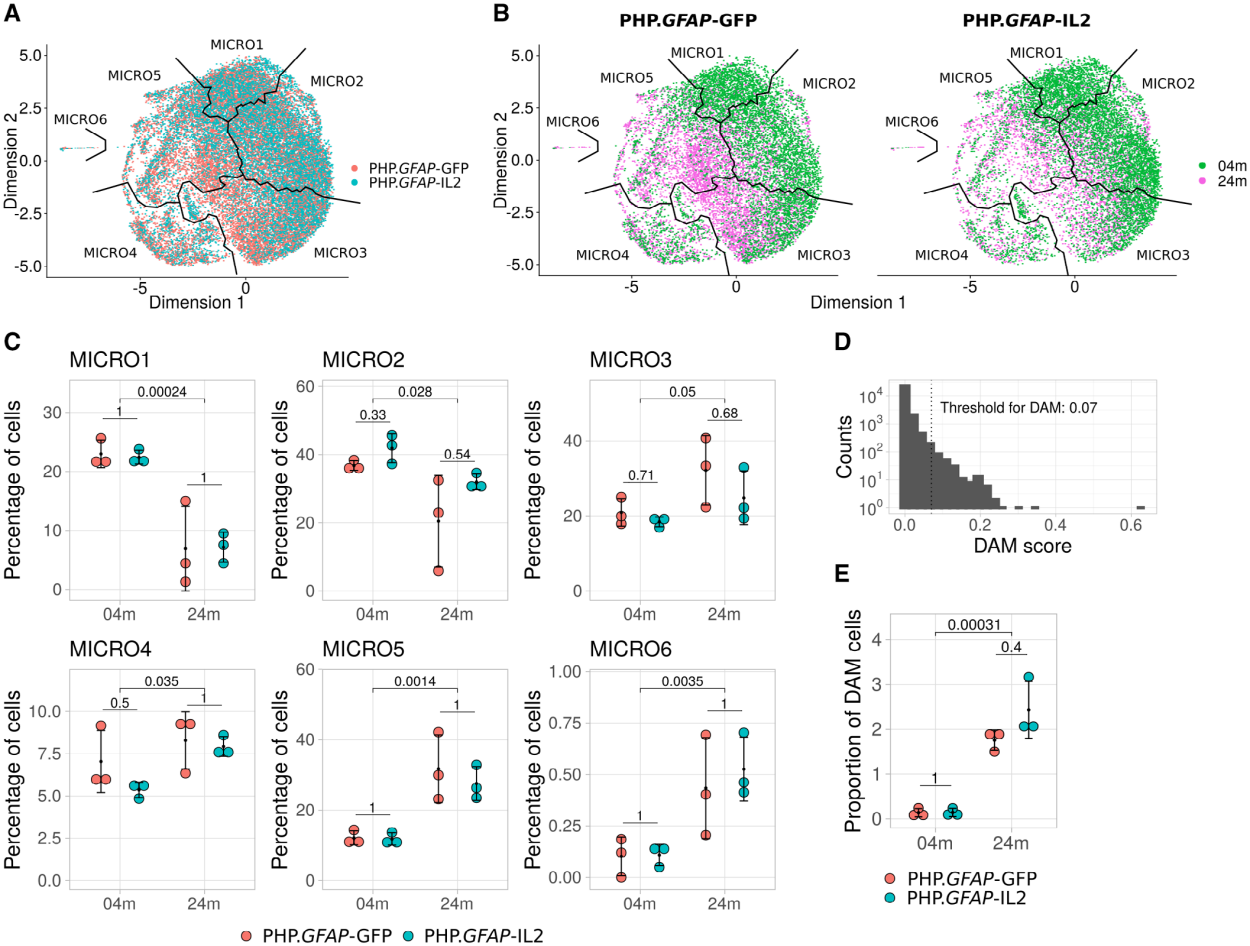
Age‐dependent accumulation of disease‐associated microglia is intact in IL2‐treated mice Wild‐type mice were treated with PHP.*GFAP*‐IL2 (or PHP.*GFAP*‐GFP control vector) at 2 months or 22 months of age (*n* = 3/group). Two‐month post‐treatment, the glial compartment was sorted from perfused mice and assessed using 10× single‐cell transcriptomics. Microglia were identified, extracted, reclustered, and reprojected in UMAP space.
Distribution of the microglia according to treatment group.Distribution of microglia by age, with subpanels separating the cells by treatment (PHP.*GFAP*‐IL2 or PHP.*GFAP*‐GFP control vector).Proportions of cells from each sample contained within each of six microglia clusters. Each panel shows multiple‐testing corrected *P*‐values for *t*‐tests comparing the means of the samples between treatments within each age group, and the means of the two age groups irrespective of treatment.Histogram of disease‐associated microglia (DAM) scoring, indicating the 0.07 threshold for classification as DAM.Proportions of DAM in each sample, with multiple‐testing corrected *P*‐values for *t*‐tests comparing the means of the samples between treatments within each age group, and the means of the two age groups irrespective of treatment. Data information: 3 biological replicates per treatment and age group (A‐E). Mean ± SD, with individual values shown (C and E). Source data are available online for this figure.

We first investigated cellular changes in the microglial compartment. Reclustered microglia were assessed for separation based on age and treatment (Fig 2A and B). Visual separation of age was observed on the UMAP plot, with quantification of clusters identifying increased representation of Micro1 and Micro2 clusters in young mice, with Micro3 to Micro6 clusters increased in aged mice (Fig 2C). Based on the prior characterization of disease‐associated microglia (DAM), we identified microglia expressing characteristic markers (Appendix Fig S4) and classified cells as DAM based on aggregate marker expression above a threshold (Fig 2D) calibrated against published databases (Keren‐Shaul *et al*, 2017). DAM were nearly absent in young mice, with a significant increase in aged mice (Fig 2E), on the order of ~ 2% of microglia. For each identified subset, no significant changes were observed due to IL2 treatment (Fig 2C and E). These data suggest that brain‐specific IL2 supplementation does not alter microglial progression through the cellular states normally observed with age.

**Figure 3:**
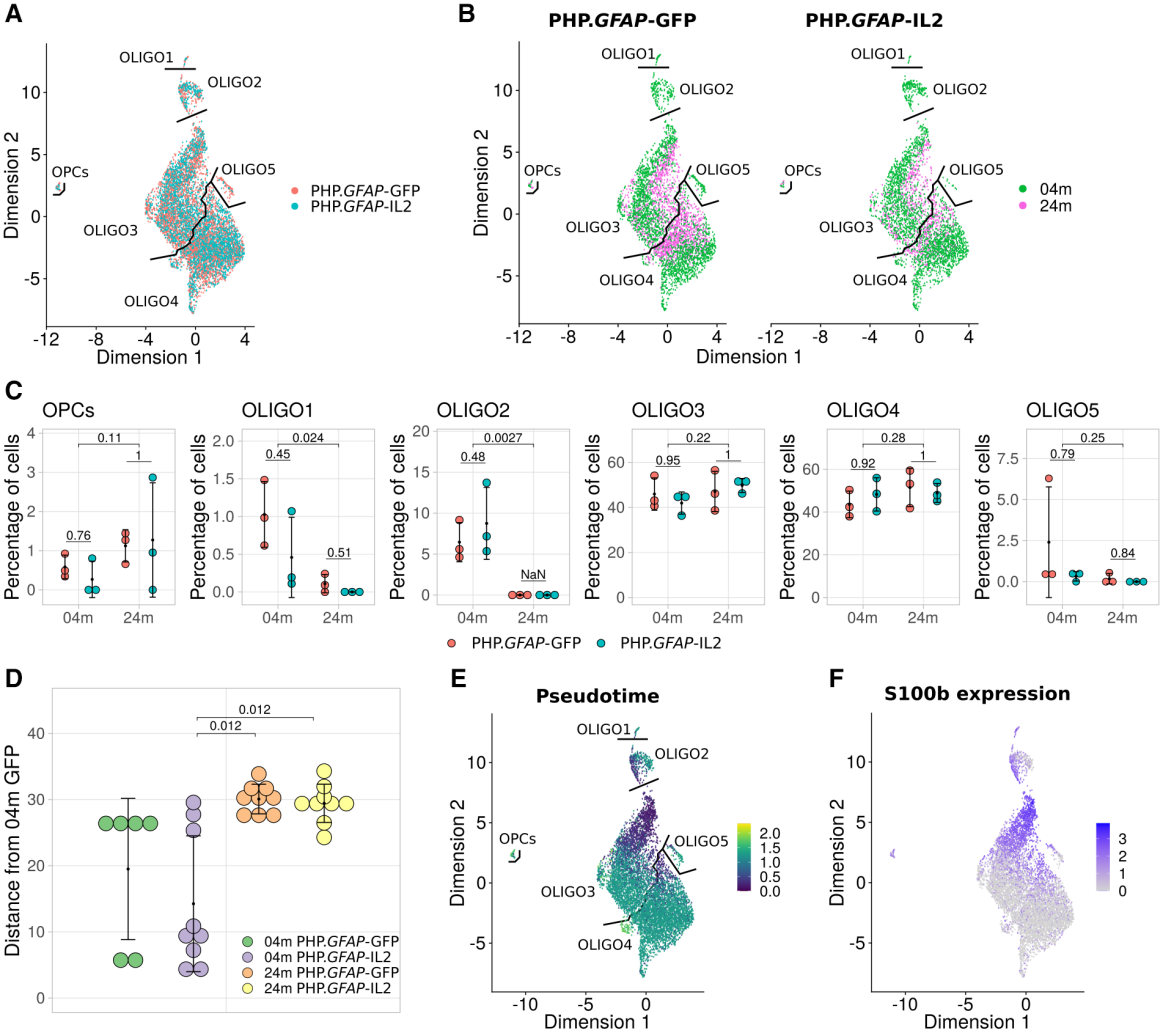
Normal age‐dependent cellular progression for oligodendrocytes following IL2‐treatment Wild‐type mice were treated with PHP.*GFAP*‐IL2 (or PHP.*GFAP*‐GFP control vector) at 2 months or 22 months of age (*n* = 3/group). Two‐month post‐treatment, the glial compartment was sorted from perfused mice and assessed using 10× single‐cell transcriptomics. Oligodendrocytes and oligodendrocyte precursor cells (OPC) clusters were identified, extracted, reclustered and reprojected in UMAP space.
Distribution of oligodendrocytes and OPCs by treatment.Distribution of the oligodendrocytes and OPCs by sample age, with subpanels separating the cells by treatment.Proportions of cells in the various oligodendrocyte and OPC clusters. Each panel shows multiple‐testing corrected *P*‐values for *t*‐tests comparing the means of the samples between treatments within each age group, and the means of the two age groups irrespective of treatment.The sum of deviance plot summarizing the proportions of age groups in the trajectory trees generated for oligodendrocytes and precursors (Fig EV2). The plot visualizes the difference in proportion of cells by age in each branch of the respective trajectory tree relative to young PHP.*GFAP*‐GFP‐treated mice, illustrating which age and treatment group shows the most deviation in the cell trajectory relative to the reference. Three biological replicates per treatment and age group. Data points are a permutation of each replicate relative to the three young control replicates.Pseudotime projection calculated using the DDRTree algorithm in Monocle v2 mapped onto the UMAP space.
*S100β* expression superimposed on the UMAP projection. Data information: 3 biological replicates per treatment and age group (A–C, E–F). Biological replicates pooled for visualization (A, B, E, F). Mean ± SD, with individual values shown (C, D). Source data are available online for this figure.

**Figure EV2:**
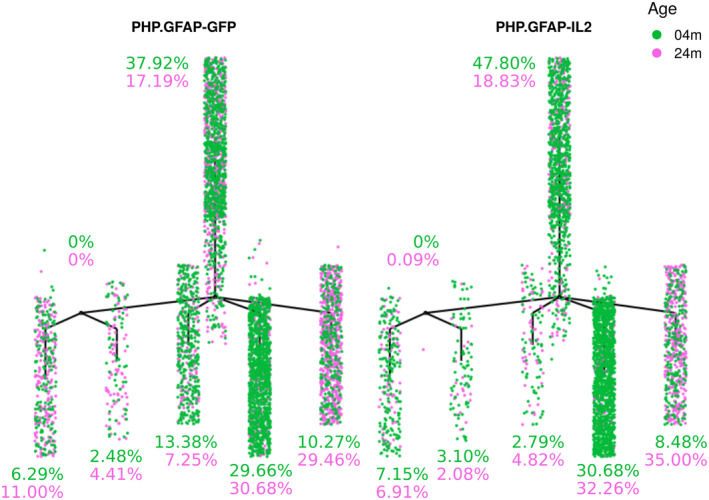
Normal pseudotime cellular trajectory progression for oligodendrocytes following IL2‐treatment Wild‐type mice were treated with PHP.*GFAP*‐IL2 (or PHP.*GFAP*‐GFP control vector) at 2 or 22 months of age (*n* = 3/group). Two‐month post‐treatment, the glial compartment was sorted from perfused mice and assessed using 10× single‐cell transcriptomics. Oligodendrocytes and OPCs were reclustered and a pseudotime trajectory constructed (branching trajectory trees), using the DDRTree algorithm in Monocle v2. The tree was rooted at the branch with the highest proportion of young cells treated with PHP.*GFAP*‐GFP. The trees were generated over the whole dataset and illustrated separately for PHP.*GFAP*‐GFP and PHP.*GFAP*‐IL2.

Reclustering of oligodendrocytes resulted in a clear separation of OPCs, with the remaining oligodendrocyte population showing a visual separation based on age (Fig 3A and B). While OPC numbers were unaffected by aging, the clusters Oligo1 and Oligo2 were reduced with age (Fig 3C). Pseudotime analysis allowed the generation of branching trajectory trees across each sample (Fig EV2). Compared with the young IL2‐treated mice, aged mice demonstrated altered proportions of cells in different stages of the pseudotime trajectory, with substantial deviations in the relative distribution of cells (Fig 3D). In concordance, pseudotime reconstruction of oligodendrocyte progression identified Oligo1 and Oligo2 clusters as the most immature state, with Oligo3 a transitional, followed by progression through Oligo4 and Oligo5 with maturation (Figs 3E and EV2). This reconstruction aligned with the expression of S100β (Fig 3F), which, in oligodendrocytes, serves as a canonical maturation marker, and other oligodendrocyte markers (Appendix Fig S5). Together, these results suggest a structured progression from immature to mature oligodendrocytes occurring in aged mice. As with microglia, brain‐specific delivery of IL2 through PHP.*GFAP*‐IL2 did not alter this normal maturational progression (Fig 3C and D).

**Figure 4:**
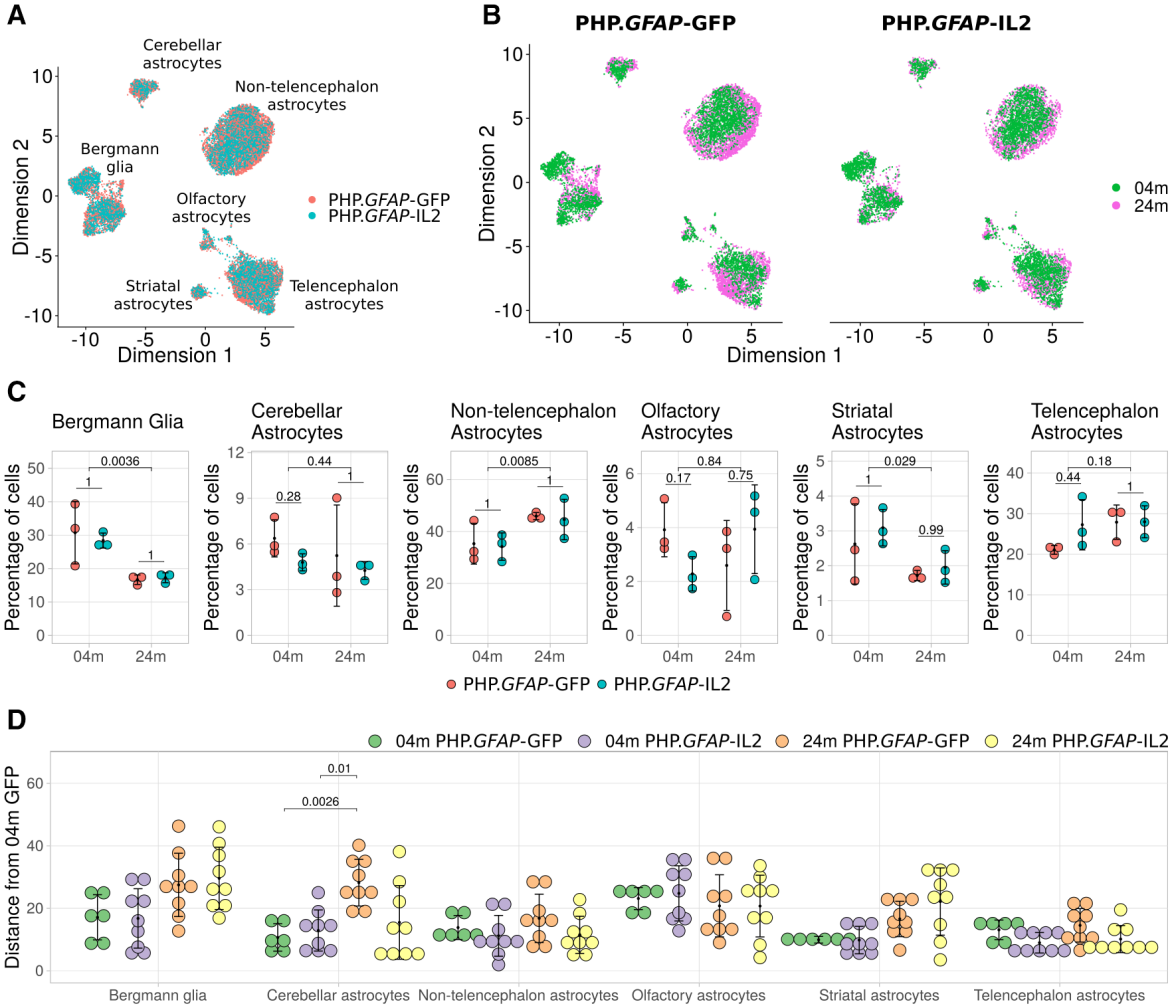
Partial prevention of age‐dependent transcriptional trajectory in astrocytes following IL2‐treatment Wild‐type mice were treated with PHP.*GFAP*‐IL2 (or PHP.*GFAP*‐GFP control vector) at 2 months or 22 months of age (*n* = 3/group). Two‐month post‐treatment, the glial compartment was sorted from perfused mice and assessed using 10× single‐cell transcriptomics. Astrocyte were identified, extracted, reclustered and reprojected in UMAP space, with subset annotation based on marker expression and mapping onto known astrocyte subtypes.
Distribution of the different astrocyte subtypes by treatment.Distribution of the different astrocyte subtypes by age, with subpanels separating the cells by treatment.Proportion of cells from each group that fall into each astrocyte subtype. Each panel shows multiple‐testing corrected *P*‐values for *t*‐tests comparing the means of the samples between treatments within each age group, and the means of the two age groups irrespective of treatment.The sum of deviance plot summarizing deviations in the proportion of cells in each branch of the pseudotime trajectory, compared to that of the young control, generated for each astrocyte subtype (Fig EV3). The plot visualizes the difference in proportion of cells by age in each branch of the respective trajectory tree relative to young PHP.*GFAP*‐GFP‐treated mice, illustrating which age and treatment group shows the most deviation in the cell trajectory relative to the reference. Three biological replicates per treatment and age group. Data points are a permutation of each replicate relative to the three young control replicates. Data information: 3 biological replicates per treatment and age group (A–C, E, F). Biological replicates pooled for visualization (A, B). Mean ± SD, with individual values shown (C, D). Source data are available online for this figure.

**Figure EV3:**
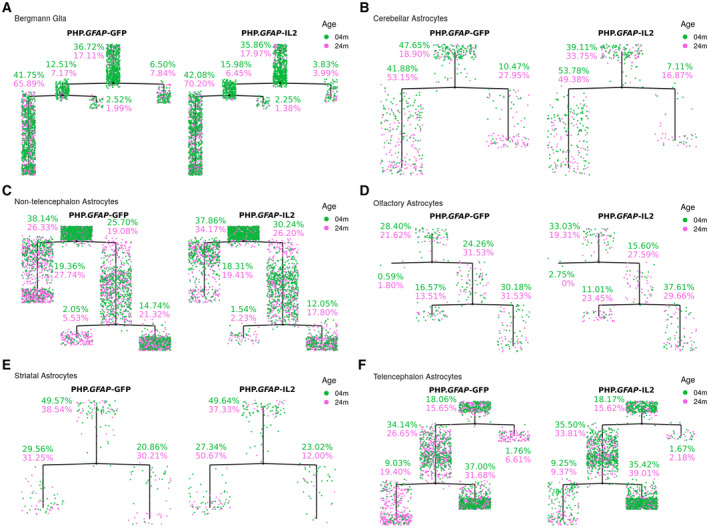
Pseudotime trajectory trees for astrocytes during aging and following treatment with IL2 Young and old wild‐type mice, treated with PHP.*GFAP*‐IL2 (or PHP.*GFAP*‐GFP control vector) were assessed 2‐month post‐treatment by single‐cell sequencing using 10x single‐cell transcriptomics. Astrocytes were identified based on marker expression and reclustered/reprojected in UMAP space. Astrocyte subclusters, annotated based on key markers, were assessed for pseudotime trajectory. Pseudotime analysis generated branching trajectory trees of cells (based on the gene expression profile of each cell), using the DDRTree algorithm in Monocle v2. The tree was rooted at the branch with the highest proportion of young cells treated with PHP.*GFAP*‐GFP.
A–F(A) The trees were generated over the whole data and illustrated separately for PHP.*GFAP*‐GFP and PHP.*GFAP*‐IL2 for Bergmann glia, (B) cerebellar astrocytes, (C) nontelencephalon astrocytes, (D) olfactory astrocytes, (E) striatal astrocytes and (F) telencephalon astrocytes.

Finally, we assessed the effects of aging and IL2 treatment on astrocyte maturation (Fig 4A). Unlike microglia and oligodendrocytes, astrocytes were highly heterogeneous, corresponding to previous anatomical separation of subsets (Batiuk *et al*, 2020; La Manno *et al*, 2021). Visual inspection of UMAP reclustering found an age‐based segregation within clusters (Fig 4B). At the subtype level, only minor shifts were observed with age—a decrease in Bergmann glia and striatal astrocytes, and an increase in nontelencephalon astrocytes (Fig 4C). To investigate the effect of aging within each subtype, we constructed pseudotime maturational trees (Fig EV3A–F). To assess the effect of age and treatment on maturation, we generated a summary statistic of the sum of frequency difference in each tree branch. Overall, aging in control‐treated groups (PHP.*GFAP*‐GFP) drove a trend toward aggregate change in Bergmann glia, cerebellar astrocytes, and striatal astrocytes (Fig 4D). Treatment with PHP.*GFAP*‐IL2 did not alter the trajectory progression in young mice; however, it partially prevented the aged trajectory distribution developing in cerebellar astrocytes (Fig 4D). Together, this analysis found substantial age‐induced transcriptome changes in all major glial cell types, with no transcriptional abnormalities caused by IL2‐delivery.

### Brain‐specific IL2 delivery mitigates age‐induced molecular changes in resident glia

**Figure 5:**
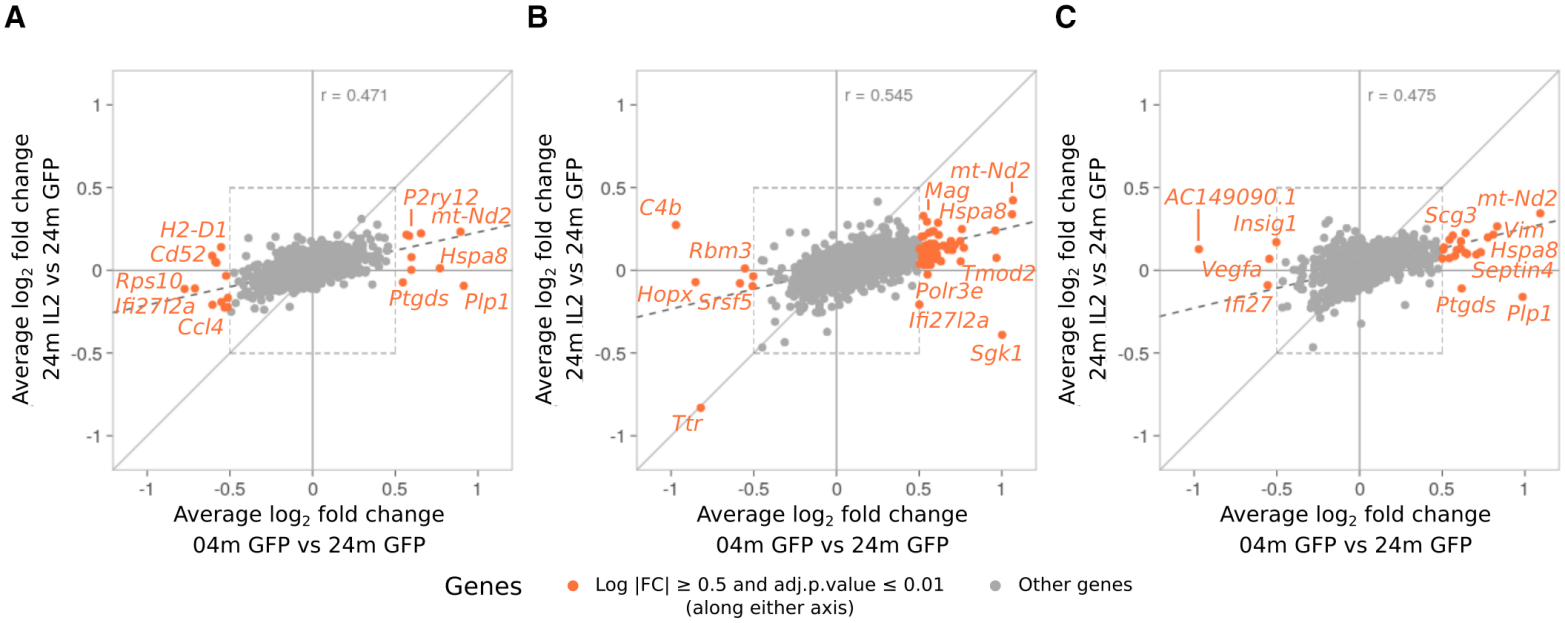
Local IL2 production in aged mice partially reverts the molecular changes occurring in aged glia A–CTwo‐dimensional differential expression plots illustrating the gene expression changes occurring during normal aging (young PHP.*GFAP*‐GFP mice versus aged PHP.*GFAP*‐GFP mice) contrasted with the changes in IL2‐treated aged mice (aged PHP.*GFAP*‐IL2 mice versus aged PHP.*GFAP*‐GFP). Expression plots shown for (A) microglia, (B) oligodendrocytes and OPCs, and (C) astrocytes (total).

**Figure 6:**
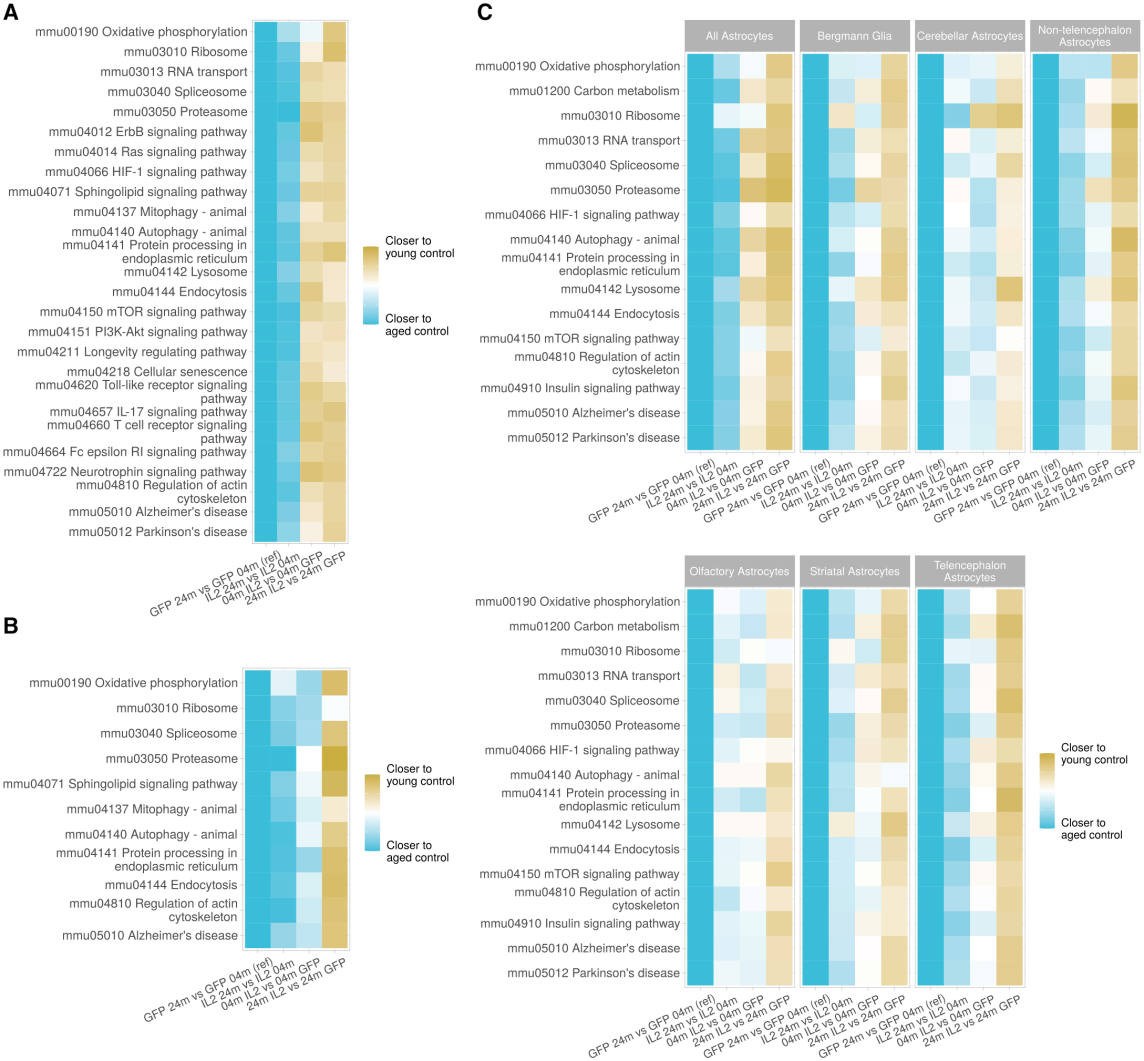
Age‐induced pathways in glia are reverted to a young transcriptional state by local IL2 production Gene set enrichment was performed based on differential expression across the comparisons for age (aged PHP.*GFAP*‐GFP mice versus young PHP.*GFAP*‐GFP mice), treatment in young mice (young PHP.*GFAP*‐GFP mice versus young PHP.*GFAP*‐IL2 mice), treatment in old mice (aged PHP.*GFAP*‐IL2 mice versus aged PHP.*GFAP*‐GFP mice) and age in treated mice (aged PHP.*GFAP*‐IL2 mice versus young PHP.*GFAP*‐IL2 mice). Gene sets were taken from the GAGE library and curated to the respective KEGG pathway. Listed pathways were manually curated for relevance to glial biology and nonredundancy. Pathways from the unbiased list were indicated if they are present in at least one of the four respective differential expression comparisons. For each pathway, directionality maps were generated, summarizing the change in direction of gene expression for member genes relative to the expression change with age (aged PHP.*GFAP*‐GFP mice contrasted with young PHP.*GFAP*‐GFP mice).
Directionality is shown for each pathway across each of the four comparisons made for microglia,Oligodendrocytes and OPCs, and.Total astrocytes and the identified astrocyte subsets.

Having established that local IL2 expression in the brain does not distort the relative frequency of major glial cell types, nor the normal trajectory distribution, we sought to investigate the impact on age‐induced molecular pathways. First, we performed a differential expression analysis for each major glial cell type (Dataset EV1). Two‐dimensional differential expression comparisons demonstrated that the largest transcriptional changes occurred with age for each glial cell type (Fig 5). The changes occurring in aged mice treated with IL2 were reduced in scale, but ran directly counter to those induced by aging alone. Correlation of comparative differential expression indicated that local IL2 treatment countered 47, 55, and 46% of the age‐induced transcriptional changes in microglia (Fig 5A), oligodendrocytes (Fig 5B), and astrocytes (Fig 5C), respectively. We next sought to determine the effect at the gene set level, where accumulated minor changes in a molecular pathway can be assessed. Gene set enrichment was performed on each glial cell type, comparing the effects of age and treatment. Among the key pathways modified by aging in microglia were those related to proteostasis and autophagy, key signaling pathways (including Ras, PI3K, and mTOR pathways), neurodegeneration‐associated genes, and the cellular senescence pathway (Dataset EV2). Oligodendrocytes and astrocytes underwent a similar transcriptional change with age, albeit with fewer pathways significantly altered, with changes in gene expression identified for pathways associated with proteostasis, autophagy, and neurodegeneration (Dataset EV2). To assess the effect of treating aged mice with local IL2 production, we created a directionality map, where each transcriptional change within a gene set was compared with the scale and direction of the change occurring with age in the control mouse. This allowed the direct comparison of transcriptional changes occurring within each pathway, across each of the glial cell types. Almost all key age‐induced pathway changes were also mirrored in IL2‐treated mice, with 26/26 pathways in microglia (Fig 6A), 10/11 pathways in oligodendrocytes (Fig 6B), and 15/16 pathways in astrocytes (Fig 6C) undergoing the same direction of transcriptional change in aged IL2‐treated mice as occurs in aged control mice. However, in almost all cases (26/26 pathways in microglia, 10/11 pathways in oligodendrocytes, and 16/16 pathways in astrocytes), IL2‐treatment of aged mice reverted the expression profile to one closer to that of the young control mice (Fig 6A–C). Together, these results demonstrate that the key coordinated transcriptional changes occurring in aged glial cells, in protein production pathways, autophagy‐associated pathways, and neurodegeneration‐associated genes, are partially reverted through a 2‐month treatment regime of locally produced IL2.

**Figure EV4:**
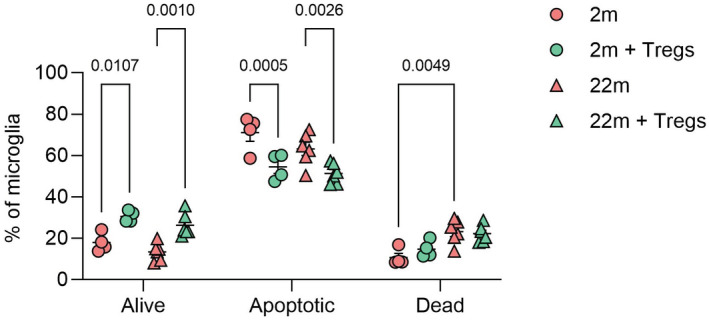
*In vitro* effects of Treg co‐culture on microglial survival Primary microglia isolated from adult or aged mice were sort‐purified and plated for 2 days, prior to co‐culturing with or without sort‐purified Tregs, supplemented with 20 ng/ml IL2, for 6 days. At the end point, microglia were assessed for apoptosis using flow cytometry, using AnnV and PI. Data information: Mean ± SEM of technical replicates; 2‐way ANOVA with age and treatment as the main factors. Representative of experiment repeated twice. Source data are available online for this figure.

To develop an *in vitro* model for the impact of IL2 and Tregs on glial cells, we used microglial cultures. While fetal microglia survive well in culture, microglia from adult mice have poor survival *in vitro*. Microglia from aged mice had higher *in vitro* death rates than those purified from adult mice (Fig EV4). The provision of IL2 and Tregs to the culture media increased microglia survival and reduced apoptosis rates (Fig EV4). While care must be taken in extrapolation from *in vitro* to *in vivo* systems, as rate‐limiting factors can vary in these contexts, these results provide a potential explanation for the molecular protection afforded by local IL2 production *in vivo*. Prevention of the aged/senescence transcriptional signature associated with local Treg expansion is consistent with long‐term (minor) reductions in apoptosis rate and cellular turnover.

### Brain‐specific IL2 gene delivery provides partial protection for cognitive decline

**Figure 7:**
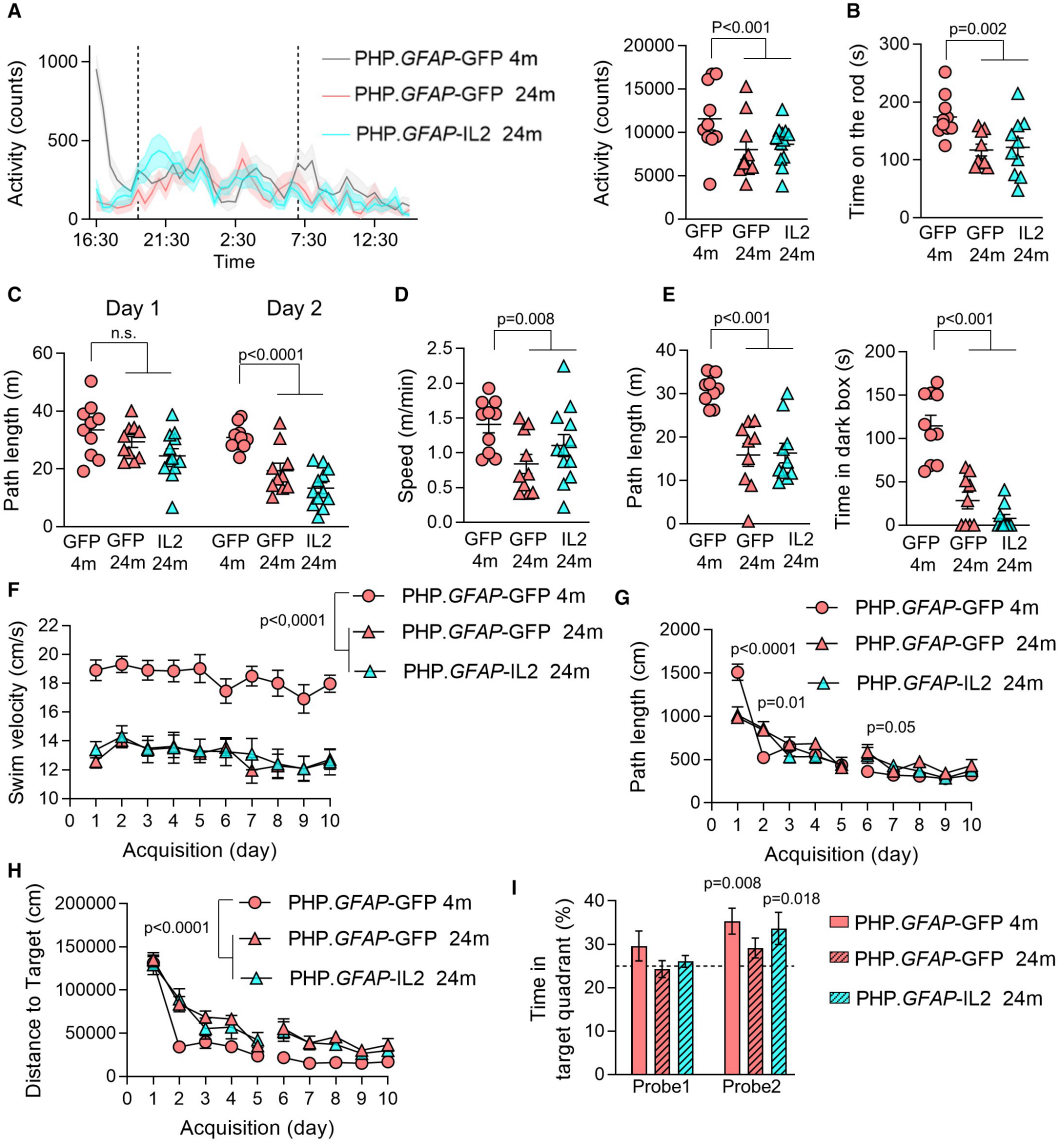
Synthetic IL2 delivery prevents age‐induced decline in spatial memory formation without altering age‐induced degeneration in mobility or novelty‐seeking behaviors Wild‐type mice treated with PHP.*GFAP*‐GFP control vector at 2 months of age were compared with aged mice treated with PHP.*GFAP*‐IL2 (or PHP.*GFAP*‐GFP control vector) at 22 months of age. Two‐month post‐treatment (4 or 24 months), behavior was tested.
AHome cage activity was assessed through infrared registration of horizontal movements of single‐housed mice every 30 min (*n* = 10, 10, 12). Left, activity over 24 h. Dotted lines indicate day/night boundaries. Right, average activity counts.BTime spent on the accelerating rotarod, average of 4 repeated tests of 300 s (*n* = 10, 9, 10).CTotal distance moved in an open field test in trials performed 1 day apart (*n* = 10, 10, 12).DExploration during the sociability test (*n* = 10, 10, 12).ELight–dark test (*n* = 10, 9, 10). Left, distance moved in the lighted arena. Right, latency to enter the dark zone.FSpatial learning in the Morris water maze (*n* = 10, 10, 12). Swim velocity.G, H(G) Path length to finding the hidden platform, and (H) distance to target during acquisition trials. The experiment was performed once, including data collection at 10 different time points (*n* = 10, 10, 12).IProbe tests after 5 days (probe 1) and 10 days (probe 2) of acquisition in the Morris water maze. The experiment was performed once, with data collection from two of the 10 different time points presented (*n* = 10, 10, 12). Time spent in the Target Quadrant was significantly above chance during the second probe trial in the young control group and in old IL2‐treated animals. Data information: Mean ± SEM. (A and F), 2‐way ANOVA repeated measures with age and treatment as the main factors, (B–D), 2‐way ANOVA with age and treatment as the main factors, (G), one‐sample *t*‐test to 25% chance level. Source data are available online for this figure.

**Figure EV5:**
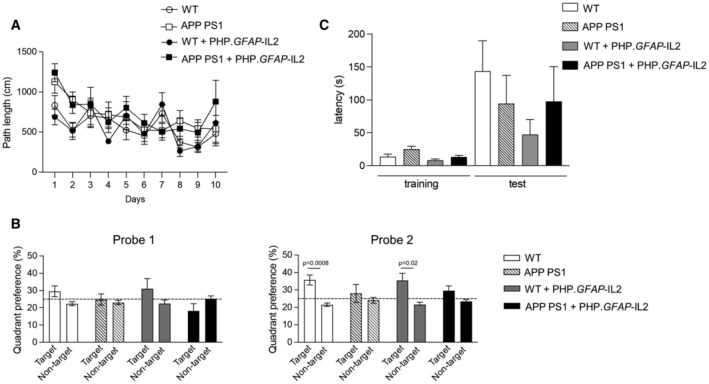
Behavioral assessment of APP PS1 mice treated with PHP.*GFAP*‐IL‐2 Female APP‐PS1 and littermate controls were administered PHP.*GFAP*‐IL2 (10^9^ vg/mouse) i.v. at 2 months of age, and behavioral tests were performed at 10 months of age.
Spatial learning in the Morris Water Maze. Path length to finding the hidden platform (*n* = 9, 7, 5, 7).Probe tests after 5 days, 10 days and after reversal learning (*n* = 9, 7, 5, 7). Dashed line represents random chance (25%) of quadrant preference.Performance in the passive avoidance test (*n* = 9, 7, 5, 7). Data information: Mean ± SEM. (B and C) 2‐way ANOVA with genotype and treatment as the main factors. Source data are available online for this figure.

Based on the ability of PHP.*GFAP*‐IL2 to partially prevent the key coordinated transcriptional signature of aging in glia, we sought to determine whether these molecular changes would result in improvements at the cognitive level. We treated old mice with PHP.*GFAP*‐IL2 or control PHP.*GFAP*‐GFP vector and initiated a battery of behavioral tests 2 months later, at 24 months of age—with a set of 4‐month‐old GFP‐treated animals used as the learning control group. Aged mice demonstrated multiple signs of behavioral decline, with reduced activity in their home cage (Fig 7A), impaired mobility on the rotarod (Fig 7B), and reduced exploration in an open field test (Fig 7C). Aged mice also demonstrated decline in the social novelty‐seeking behavior (Fig 7D) and reduced explorative behavior in the light–dark test (Fig 7E). For each of these measures, which may reflect a reduction in physical capacity and decreased arousal, brain‐specific IL2 delivery did not alter the age‐related decline. Finally, we tested spatial learning in the Morris water maze. Aged mice demonstrated poor performance in the Morris water maze in two aspects. First, aged mice had a large reduction in swim velocity during the test (Fig 7F), a phenotype likely derived from physical rather than cognitive decline. Second, aged mice showed delayed spatial learning acquisition during training (Fig 7G–I) and demonstrated a reduced preference for the target quadrant after 10 days of training (Fig 7I), a phenotype reflecting reduced cognitive capacity for spatial memory formation. Treatment of aged mice with PHP.*GFAP*‐IL2 had no impact on the physical decline in swim velocity, but was able to completely correct the defect observed in spatial memory formation (Fig 7F and H). As a preliminary test to determine whether this protective effect against cognitive decline in healthy aging could be extended to the amyloid‐driven accelerated cognitive decline observed in Alzheimer's Disease mouse models, we treated a cohort of presymptomatic APP‐PS1 mice (Jankowsky *et al*, 2004) with PHP.*GFAP*‐IL2 at 2 months of age. At 10 months of age, APP‐PS1 transgenic mice exhibited cognitive decline, with elevated pathlength (Fig EV5A) and poor quadrant preference (Fig EV5B) in the Morris Water Maze and poor latency in the passive avoidance test (Fig EV5C). This poor performance was not improved by PHP.GFAP‐IL2 treatment (Fig EV5A–C). While not excluding potential efficacy at different doses or treatment regimes, these results suggest the IL2 treatment is less efficacious in amyloid‐driven cognitive decline than in age‐related cognitive decline. Together, these results suggest that PHP.*GFAP*‐IL2, even when delivered to old mice, is able to prevent or restore cognitive decline in spatial memory formation, without impacting other aspects of age‐related behavioral decline, such as physical prowess and arousal.

## Discussion

Brain aging is a multifaceted phenomenon, encompassing structural, functional, and microenvironmental changes that impact on both the neuronal and glia compartments. However, the causative drivers of deleterious cellular changes are difficult to untangle, as alterations such as loss of neuronal circuits and plasticity could be primarily driven by cell‐intrinsic neuronal mechanisms of aging or alternatively be derived from the impact of aging glial support. The role of inflammation in this process is likely to be a primary driver of at least some aspects of the aging process, based on correction experiments. For example, defects in learning and memory capacity in the contextual fear conditioning and radial arm water maze assays can be partially corrected in 18‐month‐old mice through the repeated injection of plasma from young mice (Villeda *et al*, 2014). Likewise, heterochronic parabiosis, joining the circulatory system of a 21‐month‐old mouse to a 2‐month‐old mouse, improved the performance of old mice in olfactory sensitivity assays (Katsimpardi *et al*, 2014). Thus, while a neuronal cell‐intrinsic aging effect is likely, an emergent feature of cognitive decline must at least partly depend on environmental factors, such as a build‐up of toxic or inflammatory products. T cells are a key potential player in the development of this neuroaging environment. T cells can directly produce or stimulate the production of, key inflammatory mediators, including cytokines, ROS and autoantibodies, a process that can directly contribute to emergent features such as the constraint of the neuronal stem cell niche (Dulken *et al*, 2019). In particular, IFNγ production has emerged as a potential mediator of age‐related decline, with activation of microglia and astrocytes leading to neuronal damage (Liston & Yshii, 2023). The identification of T cells as a potential culprit opens the possibility of Tregs as a therapeutic. Tregs have direct anti‐inflammatory properties, preventing excessively exuberant responses from conventional T cells. The protective effects observed here could potentially be driven through direct countering of T cell‐mediated inflammation, although this modality of control would be restricted due to low densities of both cell types within the brain (Pasciuto *et al*, 2020). In addition, Tregs can produce pro‐repair‐orientated cytokines, such as amphiregulin and osteopontin (Ito *et al*, 2019; Shi *et al*, 2021), which are neuroprotective following injuries such as stroke. As Treg numbers in the brain are low, it is expected that their influence will be mediated, at least in part, through the reprogramming of glial cells. In the peripheral context, Tregs are capable of reprogramming monocytes into the more pro‐repair anti‐inflammatory profile (Tiemessen *et al*, 2007). In the brain, an analogous program may be imparted onto microglia (Zhou *et al*, 2017), and our results here indicate potential for Tregs to slow cellular turnover of microglia, which may delay senescence. Microglial polarization can, in turn, impact astrocyte polarization (Li *et al*, 2020). Tregs can also stimulate OPC maturation into oligodendrocytes, promoting myelination, in a pro‐repair state (Dombrowski *et al*, 2017). When considering the efficacy of PHP.*GFAP*‐IL2 in mitigating aging effects, the most parsimonious explanation would be a primary effect via the local expansion of Tregs. When used in the context of traumatic brain injury, the beneficial effects of PHP.*GFAP*‐IL2 were only observed in the presence of an adaptive immune system, suggesting that the levels of IL2 achieved (~ 2 pg/ml) are too low to trigger the activation of the lower affinity receptor expressed in non‐Treg lineages (Yshii *et al*, 2022b). While it would be attractive to speculate on the role of individual downstream mediators, the complexity and interdependencies of aging make a multifactorial function more likely, which integrate pro‐repair, anti‐inflammatory, and glial reprogramming functions. The *in vitro* system developed here may allow future dissection of some of these mechanisms.

Single‐cell transcriptomics of aging glia identified a shared age‐induced molecular signature across all major glial cell types. The pathways identified concord with our current molecular understanding of aging, led at the cellular level by mitochondrial dysfunction, loss of proteostasis, and cellular senescence. Mitochondrial dysfunction (here identified as changes in the oxidative phosphorylation and mitophagy pathways) has been identified as a key cell‐intrinsic marker of aging, with dysfunction in microglia a potential contributor to neurodegeneration in dementia (Chakravorty *et al*, 2019). Indeed, an increased burden of mitochondrial dysfunctional can hasten the progression of brain atrophy in mouse models of AD (Kukreja *et al*, 2014), while mitophagy of defective mitochondria reverses cognitive decline (Fang *et al*, 2019). Loss of proteostasis is another reoccurring theme in aging across tissues and is consistent here with changes to protein production (RNA transport, spliceosome, and ribosome), processing (protein processing in endoplasmic reticulum), and degradation (proteasome) pathways. The mechanistic link between proteostasis and aging is unclear, with a lead contender being the toxic or inhibitory build‐up of misfolded and/or damaged proteins. The genetic link between proteostasis network components and neurological diseases, such as ALS, AD, and PD, strongly suggests that a failure of normal proteostasis is a distinct risk factor for neuropathology (Labbadia & Morimoto, 2015). Related to proteostasis is autophagy, a process intimately linked to aging (Aman *et al*, 2021). AD patients exhibit dysregulated autophagy (Lee *et al*, 2010) and loss of autophagy causes severe neurodegeneration in mice (Komatsu *et al*, 2006). For the specific signaling pathways identified, the Neuregulin‐ErbB pathway, Ras pathway, and PI3K/AKT/mTOR (Borras *et al*, 2011; Mazucanti *et al*, 2015; Ou *et al*, 2021) are all integral to neuroaging and potential drivers for the cellular phenotypes developing in aging glial cells.

Surprisingly, the local provision of IL2 in the brain was sufficient to substantially revert almost all of these age‐induced pathway changes across microglia, oligodendrocytes, and astrocytes. This was despite relatively few immune pathways being identified as altered, apart from the upregulation of MHCII, previously linked to PHP.*GFAP*‐IL2 treatment (Yshii *et al*, 2022b), and alterations in the TLR and IL17 signaling pathways in microglia. Whether this effect is mediated via direct reprogramming, such as Treg cytokine‐mediated effects, or indirect effects of microenvironmental cleansing, remains to be seen. It is, however, highly promising that such cell‐intrinsic molecular signatures of aging are responsive to late‐stage intervention.

While this study suggests that an analog of the PHP.*GFAP*‐IL2 treatment could be of use to avert cognitive decline during human aging, there are several key barriers to translation. First, the degree to which the aging process is conserved across the species barrier is unclear. Many of the biological processes occurring in the aged brain, such as accumulation of DNA modifications, mitochondrial dysfunction, and loss of proteostasis, are shared across species (Yankner *et al*, 2008). Likewise, cognitive decline is common between mice and humans (Gallagher & Rapp, 1997), including reduction in spatial navigation performance (such as the Morris water maze in mice, or the equivalent in humans; Moffat *et al*, 2007). Despite this, mouse cognitive decline seems to appear relatively sooner and faster than for humans (Yanai & Endo, 2021), and decline in elaborate cognitive abilities specific to human, such as language, cannot be tested in animal models. Further, the neurogenic niche inhibited by T cells in aged mice (Dulken *et al*, 2019) may not exist in humans (Franjic *et al*, 2021). Thus, even if the reversal of impaired spatial navigation in humans was possible through brain‐specific IL2 delivery, it is not clear that other aspects of cognitive decline, such as language deficits, which potentially share a common cellular or molecular cause, would necessarily respond to the same treatment approach. A second potential limitation to translation is the kinetics involved in cognitive decline. Here, we treated aged mice for 2 months prior to assessment, with the treated aged mice performing similar in spatial navigation capacity to young control mice. It remains unknown, however, whether the treatment actively reversed the molecular, cellular, and behavioral processes of aging, or whether it merely prevented the decline. The kinetics of cognitive decline we measured are compressed into a mouse lifespan, raising the question of whether treatment in humans could impact a decline occurring over the course of decades. If cognitive decline is primarily driven by active changes in the brain microenvironment, such as increased basal inflammation induced by a lifelong low‐level activation of the immune system (Montecino‐Rodriguez *et al*, 2013; Deleidi *et al*, 2015), then even transient pulses of anti‐inflammatory treatment could reverse or prevent the decline. On the contrary, if cognitive decline is driven primarily by programmed senescence, then such a treatment could be expected to, at most, delay cognitive decline. Fortunately, experiments in mice largely support the former model over the latter, as murine cognitive decline occurs with age even though individual mouse neurons can long out‐survive a mouse, when transplanted into a longer‐lived host (Magrassi *et al*, 2013), although does not exclude irreparable functional decline. A third limitation lies in the heterogeneity of causes for cognitive decline. Similar cognitive manifestations can develop from distinct molecular and cellular pathways impairing neurological networks. The degree to which the process is driven by inflammation is likely to vary from individual to individual, even within the aged population. Potential response to an IL2‐based therapy will likely vary, as illustrated, for example, by the data shown here on protection from cognitive decline in a pure age‐based model but not in a model driven by amyloid plaque formation. Finally, the molecular basis of the treatment itself needs to be considered. The delivery system used here, the PHP.B capsid, performs poorer in blood–brain‐barrier crossing in nonhuman primates than it does in mice (Hordeaux *et al*, 2018). Fortunately, alternative AAV capsids are available that perform well in humans (Deverman *et al*, 2018), with active research on improving neurodelivery via capsid design (Chen *et al*, 2022; Goertsen *et al*, 2022), and sustained cargo expression in the brain has been observed to span years (Mittermeyer *et al*, 2012), an advantage for a potential longevity treatment. Tregs are found in both the mouse and human brain (Pasciuto *et al*, 2020), and the IL2 pathway is highly conserved across the species, so it is likely that the expansion of brain Tregs could be induced in humans if cargo delivery was achieved. Nonetheless, substantial technical and regulatory barriers exist when considering the development of any longevity treatments that are designed for use in otherwise healthy individuals, with long‐term safety data being essential.

## Materials and Methods

### Mice

Foxp3^Thy1.1^ reporter mice (Liston *et al*, 2008) were used on the C57BL/6 background. APP‐PS1 mice (Jankowsky *et al*, 2004) were used on the C57BL/6 background. Wild‐type C57BL/6 mice at 2 and 22 months of age were purchased from Janvier Labs and housed in SPF conditions, under a 12‐h light/dark cycle in a temperature and humidity‐controlled room with *ad libitum* access to food and water. All animal procedures were approved by the KU Leuven Animal Ethics Committee (P124/2019), taking into account relevant national and European guidelines. Male and female mice were used in this study, unless otherwise specified. Sample sizes for mouse experiments were chosen based on power calculations and pilot data, in conjunction with the Animal Ethics Committee, to allow for robust sensitivity without excessive animal use. Mice were selected randomly for inclusion into the various experimental groups, with the animal technicians performing experimental procedures and clinical measurements blinded as to the identity of experimental groups. No mice were excluded from analysis.

### Behavioral experiments

Behavioral experiments for wild‐type mice were performed in 4‐ and 24‐month‐old males and compared with littermate controls. Behavioral experiments for APP‐PS1 mice were performed at 10 months and compared with littermate controls. Mice were habituated to their new environment for at least 5 days, and tests were conducted during the light phase of their activity cycle. Tests were performed and analyzed by an observer blind to the genotype and/or treatment group. If tracking error(s) occurred, mice were excluded.

#### Cage activity

Ambulatory behavior was investigated over a 23‐h time period, starting at 4 p.m. until 3:30 p.m. the following day (Verreet *et al*, 2016). Lights were switched off at 7 p.m., and on again at 7 a.m. During this test, animals were individually housed in transparent cages (20 × 26 cm) with chow, water, and minimal bedding and placed in a laboratory‐built activity logger with three infrared beams. Infrared beams register horizontal movements; beam breaks were recorded over 30‐min time bins.

#### Open field

Open field exploration was tested in a 50 cm × 50 cm × 30 cm (w × l × h) square arena illuminated by indirect light. Animals were dark‐adapted for 30 min and tested in the arena for 10 min. Mouse movements were video‐tracked for 10 min using ANY‐maze Video Tracking System software (Stoelting Europe).

#### Light/dark test

The apparatus used for the light/dark test consisted of a cage (50 × 50 × 30 cm) divided into two compartments by a partition with a door. One compartment was brightly illuminated, while the other was dark. Mice were placed in the illuminated compartment and allowed to move freely between the two chambers for 10 min. Mouse activity in the illuminated compartment was tracked using ANY‐maze Video Tracking System software (Stoelting Europe).

#### Rotarod test

Motor coordination and equilibrium were tested with a rotarod setup (MED Associates Inc.). Mice were first trained at a constant speed (4 rpm, 2 min) before undergoing four test trials (intertrial interval, 10 min), during which rotarod speed was increased from 4 rpm to 40 rpm over a period of 300 s. Latency to fall off the rod was recorded, up to the 5‐min cutoff point.

#### Morris water maze (MWM)

Spatial learning and cognitive flexibility were tested in the hidden platform Morris water maze (MWM). A circular pool (150 cm diameter) was filled with opacified (0.01% Acusol OP301, Dow Chemicals) water (26 ± 1°C). The platform (15 cm diameter) was hidden 1 cm underneath the surface of the water. For spatial learning, the mice were trained for 10 days to navigate to a fixed platform position (D'Hooge & De Deyn, 2001; Callaerts‐Vegh *et al*, 2006). To evaluate reference memory, probe trials (100 s) were conducted on days 6 and 11 during acquisition learning. During probe trials, floater mice were excluded. The escape platform was removed from the pool, and mice were allowed to explore the maze for 100 s. Swim paths were tracked with Ethovision software (Noldus).

#### Sociability test

Sociability was evaluated using the three‐chamber test. The setup consisted of a rectangular transparent Plexiglas box divided into three compartments, separated by two partitions. Multiple holes in the partitions allowed sniffing interactions between a mouse in the central chamber (42 × 26 cm) and a mouse in either the left or right chamber (26 × 26 cm). The test consisted of two consecutive stages: an acclimatization stage and a sociability stage. After habituation to the central compartment (5 min), a stranger mouse (same sex) was placed in one of the side chambers, while the other was left empty. Approach behavior to the side compartments was then recorded for 10 min (sociability). Animal behavior was recorded using a webcam and ANY‐maze Video Tracking System software (Stoelting Europe).

#### Passive avoidance

Dark‐adapted animals were placed in a brightly illuminated box and allowed to enter a dark compartment fitted with an electrifiable grid. Time to enter was recorded (train). When animals had entered the dark compartment, the door was closed and they received a mild foot shock (0.3 mA, 2 s) before they were replaced in their home cage. The experiment was repeated with latency to enter the dark compartment recorded (test), up to a cutoff point of 5 min.

### *In vitro* cell death assay

Primary murine microglial cells were isolated from adult (P240) and aged (P660) wild‐type female mice as described previously (Lopez‐Serrano *et al*, 2019). Forebrains were harvested and placed in cold HBSS, mechanically triturated and enzymatically dissociated using the Neural Tissue Dissociation Kit (P) (Miltenyi), following the manufacturer's specifications. Freshly dissociated cells were passed through a 70 μm cell strainer (BD2 Falcon) and washed with MACS buffer (Miltenyi). Cells were incubated with CD11b microbeads and passed through a LS column (QuadroMACS, Miltenyi). Murine microglia were plated in 24 well plates at a density of 50,000 cells/well, and cultured using TIC medium (DMEM/F12), glutamine (2 mM), N‐acetyl cysteine (5 mg/ml), insulin (1:2,000), apo‐transferrin (100 mg/ml), sodium selenite (100 ng/ml), cholesterol (1.5 mg/ml), heparan sulfate (1 mg/ml) supplemented with 50 ng/ml M‐CSF, 100 ng/ml IL‐34, 10 ng/ml CX3CL1, and 2 ng/ml TGFβ (Bohlen *et al*, 2017; Mancuso *et al*, 2019).

Two days after plating, media of both control and co‐cultured cells were supplemented with recombinant IL‐2 (20 ng/ml, BioLegend) and primary regulatory T cells. Regulatory T cells were purified from a single‐cell suspension obtained from the lymph nodes and spleen of Foxp3^Thy1.1^ reporter mice based on CD4 enrichment, using a MojoSort™ Mouse CD4 T Cell Isolation Kit (BioLegend), according to the manufacturer's instructions. Cell sorting was performed using a Sony MA900 cell sorter equipped with a 100 μm nozzle, using an antibody panel including eBioscience™ Fixable Viability Dye eFluor™ 780 (1:2,000), CD4 (AB_312696; 1:200) and Thy1.1 (AB_465773; 1:200) (for Foxp3). Sorted cells were collected in DMEM supplemented with 10% FBS. Postsort, the cell count and the viability of the samples were confirmed using a LUNA‐FL dual fluorescence cell counter (Logos Biosystems). Regulatory T cells were added to microglia cultures at a density of 50,000 cells/well. Cells were cultured for 5 days before analysis. Before harvesting, cells were washed with PBS, followed by recovery in 5 mM EDTA‐PBS using a cell scrapper. Cell death and apoptosis in microglia was measured using an annexin V (AnnV) and propidium iodide (PI) dual staining strategy (Dead cells apoptosis kit, Invitrogen). Harvested cells were washed with assay buffer and incubated with APC‐conjugated AnnV (AB_2575165; 1:50), PI and anti‐CD11b‐BV421 (AB_11203704; 1:1,000), following the manufacturer's instructions. Stained cells were analyzed by flow cytometry on a FACSCanto II cytometer (Becton Dickinson). The experiment was repeated on two independent occasions.

### AAV vector production and purification

AAV‐PHP.B production was performed by Vigene Sciences (Rockville, MD, USA), using the classical tri‐transfection method, with subsequent vector titration performed using a qPCR‐based methodology (Rincon *et al*, 2018; Fripont *et al*, 2019). For AAV‐PHP.B.*GFAP*‐IL2, the mouse IL2 coding sequence, together with 5′ and 3′ UTR (accession number BC116845), was cloned into a single‐stranded AAV2‐derived expression cassette, containing a full‐length GFAP promoter (Brenner *et al*, 1994), woodchuck hepatitis post‐transcriptional regulatory element (WPRE) and bovine growth hormone polyadenylation (bGH polyA) sequence. Control vectors were prepared by swapping the IL2 coding sequence for that encoding enhanced green fluorescent protein (EGFP; Vector Biolabs).

### AAV treatment

AAV vector (100 μl total volume) was administered to mice via the lateral tail vein at 1 × 10^9^ vector genomes/dose. Wild‐type mice were used for experimental procedures 2 months after AAV injection, unless otherwise indicated. APP‐PS1 mice were treated at 2 months of age and assessed for behavioral changes at 10 months of age.

### Flow cytometry

Single‐cell suspensions from brain were prepared using digestion at 37°C with a mixture of 1 mg/ml collagenase IV (Thermo Fisher), 300 μg/ml hyaluronidase (Sigma‐Aldrich) and 40 μg/ml DNase I (Sigma‐Aldrich) in RPMI 1640 supplemented with 2 mM MgCl_2_, 2 mM CaCl_2_, 20% FBS and 2 mM HEPES pH 7.4 (Gibco) for 30 min. This was followed by mechanical disruption, filtration, and enrichment for leukocytes by gradient centrifugation (40% Percoll GE Healthcare). Cells were fixed and permeabilized with the eBioscience Foxp3 staining kit (eBioscience) as previously described (Whyte *et al*, 2022). Cells were stained for Foxp3 (AB_2651768; 1:200), CD4 (AB_2722549; 1:500), CD3 (AB_11218085; 1:5,000), CD45 (BB790 custom order; 1:2,000), TCRβ (AB_493344; 1:2,500), and CD25 (AB_2739522; 1:1,000). Data were acquired on a BD Symphony and compensated using AutoSpill (Roca *et al*, 2021).

### Statistics

Comparisons between two groups were performed using unpaired two‐tailed Student's *t*‐tests. *Post hoc* Holm's or Dunnett's multiple comparisons tests were performed, when required. Two‐way ANOVA was used when appropriate, with age and treatment as the main factors. Cage activity behavior and MWM acquisition was analyzed with 2‐way ANOVA with repeated measures, with age and treatment as the main factors. Nonparametric testing was performed when data were not normally distributed (QQ plot for visual check and Shapiro–Wilk normality test on pooled residuals). The value of *n* reported within figure legends represents the number of animals, unless otherwise specified. Values are represented as mean ± SEM.

### Single‐cell RNA sequencing

Mice were deeply anesthetized with an intraperitoneal injection of a ketamine (87 mg/kg)/xylazine (13 mg/kg) mixture and transcardially perfused with ice‐cold PBS. The brains were put into brain medium: DMEM (Gibco) supplemented with B27 (Thermo Fisher), 2 mM sodium pyruvate (Gibco), 500 μM N‐acetyl‐cysteine (Sigma‐Aldrich) and 5 uM actinomycin D (Sigma‐Aldrich). The brains were then cut into small pieces and digested in brain medium using 33 UI/ml papain (Sigma‐Aldrich) and 40 μg/ml DNase I (Sigma‐Aldrich) for 30 min at 37°C. Digested tissue was mechanically disrupted, filtered through 100 μm mesh and enriched by density centrifugation (300 *g*_Av_, 11 min, no brake) through 24% Percoll (GE Healthcare). Cell sorting was performed using a Sony MA900 with a 100 μm nozzle, using an antibody panel including eBioscience™ Fixable Viability Dye eFluor™ 780 (1:1,000), CD11b (AB_953558; 1:400), CD45 (AB_465668; 1:1,000), CX3CR1 (AB_2565706; 1:200) (for microglia), O4 (AB_2751960; 1:100) (for oligodendrocytes), ACSA‐2 (AB_2727427; 1:100) (for astrocytes) and PDGFRα (AB_466607; 1:50) (for OPCs) for sorting. Specific cell populations were recovered and resuspended in DMEM with 10% FBS. Cell count and viability of the samples were immediately confirmed using a LUNA‐FL dual fluorescence cell counter (Logos Biosystems). Postcell count and quality control, the samples were loaded onto a Chromium Controller. For each experiment, approximately 8,700 cells were added to each channel for a targeted cell recovery of 5,000 cells. Library preparations were performed using the 10× Genomics Chromium Single Cell 3′ Kit, v3 (10× Genomics). Libraries were prepared according to the manufacturer's instructions (Single cell 3′ reagent kits v3 user guide; CG000183 Rev C), and at the different recommended check points library quality was assessed using a Qubit 2 Fluorometer (ThermoFisher) and a Bioanalyzer HS DNA kit (Agilent). With a sequencing coverage targeted for 50,000 reads per cell, single‐cell libraries were sequenced on an Illumina Novaseq 6000 or Illumina HiSeq platform using a paired‐end sequencing workflow with recommended 10X, v3 read parameters (28‐8‐0‐91 cycles).

Fastq from the raw sequencing data underwent quality control (QC) analysis and was converted to counts data using the 10× Genomics CellRanger v4.0.0 counts pipeline (Zheng *et al*, 2017). QC parameters were found to be within the expected ranges for single‐cell sequencing data, as per 10× Genomics user guides. The counts data were further analyzed using Seurat v4.0.2 (Hao *et al*, 2021) in R v4.0.5 (R Core Team, 2021). The data were filtered to remove low‐quality cells, classed as those containing > 10% mitochondrial genes (Osorio & Cai, 2021). The individual counts data were then combined and normalized by the variance stabilizing transformation method, using the sctransform v0.3.2 library (Hafemeister & Satija, 2019), correcting for batch and regressing for RNA counts and percentage mitochondrial gene expression. The normalized data were then analyzed for detection and removal of multiplets using scDblFinder v1.4.0 (Germain *et al*, 2021).

Identification of population markers and analysis of differential gene expression were performed using the built‐in methods in Seurat, using the negative binomial test. Likewise, PCA and UMAP projections were also performed using Seurat. External data were downloaded directly from the Mouse Brain Atlas (mousebrain.org/downloads; La Manno *et al*, 2021) for mapping onto the astrocyte data generated in this study, using the data integration pipeline in Seurat. Pseudotime analyses were performed and trajectories were constructed using the DDRTree algorithm in Monocle v2.22.0 (Qiu *et al*, 2017). Gene set enrichment and pathway enrichment were performed using GAGE v2.44.0 (Luo *et al*, 2009) and Pathview v1.34.0 (Luo & Brouwer, 2013), respectively.

## Author contributions

**Pierre Lemaitre:** Formal analysis; investigation; methodology; writing – original draft; writing – review and editing. **Samar HK Tareen:** Data curation; software; formal analysis; investigation. **Emanuela Pasciuto:** Data curation; formal analysis; supervision; validation; investigation; methodology; writing – original draft; project administration; writing – review and editing. **Loriana Mascali:** Investigation. **Araks Martirosyan:** Software; investigation. **Zsuzsanna Callaerts‐Vegh:** Investigation; methodology; writing – review and editing. **Suresh Poovathingal:** Investigation. **James Dooley:** Conceptualization; supervision; writing – review and editing. **Matthew Holt:** Conceptualization; supervision; funding acquisition; writing – original draft; project administration; writing – review and editing. **Lidia Yshii:** Conceptualization; supervision; validation; investigation; methodology; writing – original draft; project administration; writing – review and editing. **Adrian Liston:** Conceptualization; supervision; funding acquisition; writing – original draft; project administration; writing – review and editing.

## Disclosure statement and competing interests

The VIB and Babraham Institute have filed a patent application covering aspects of the work included in the publication and are pursuing commercialization. The authors are potential financial beneficiaries of this commercialization effort. S.T. is currently an employee of Roche.

## For more information

https://www.braincouncil.eu: network of patient organizations, scientific societies, professional societies and industry partners.
https://www.listonlab.uk/: home‐page for the Liston lab.
https://www.i3s.up.pt/research‐group.php?groupid=201: home‐page for the Holt lab.

## Supporting information



Appendix

Expanded View Figures PDF

Dataset EV1

Dataset EV2

Source Data for Expanded View

PDF+

Source Data for Figure 2

Source Data for Figure 3

Source Data for Figure 4

Source Data for Figure 7

## Data Availability

The full analysis code is available on GitHub (https://github.com/AdrianListon/AAV‐IL2) with the data available on GEO as dataset GSE190486 (http://www.ncbi.nlm.nih.gov/geo/query/acc.cgi?acc=GSE190486).
