# Supplementary material for: Molecular and cognitive signatures of ageing partially restored through synthetic delivery of IL2 to the brain
Source: EMBO Mol Med. 2023 Mar 28;15(5):e16805. doi: 10.15252/emmm.202216805 (PMC10165365; doi:10.15252/emmm.202216805)

Appendix

Appendix Figure S1……………………………………………………………………2
Appendix Figure S2……………………………………………………………………3
Appendix Figure S3……………………………………………………………………4
Appendix Figure S4……………………………………………………………………6
Appendix Figure S5……………………………………………………………………8

Appendix Figure S1. Identification of major glial populations based on key transcriptional markers. Young and old wildtype mice, treated with PHP.*GFAP*-IL2 (or PHP.*GFAP*-GFP control vector) were assessed two months post-treatment by single cell sequencing of isolated glial cells using 10x single-cell transcriptomics. UMAP feature expression plots for key microglial markers (*Tmem119*, *P2ry12*, *Cx3cr1*, *Hexb*), oligodendrocyte markers (*Mog*, *Cldn11*, *Mobp*, *Mbp*), astrocyte markers (*Aqp4*, *Fgfr3*, *Slc4a4*, *Sox9*) and oligodendrocyte precursor cell (OPC) markers (*Pdgfra*, *C1al1*, *Olig2*, *Neu4*). Color indicates expression level.

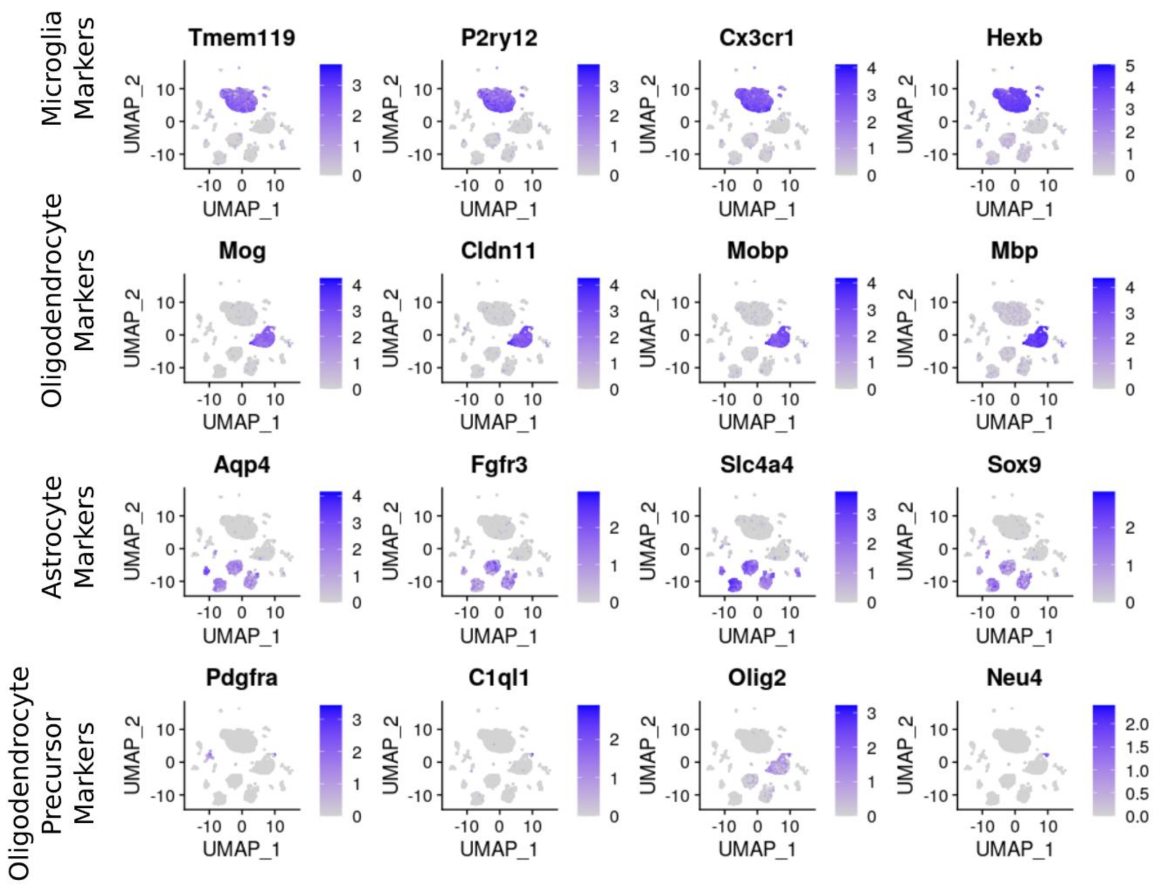

Appendix Figure S2. Identification of non-target populations based on key transcriptional markers. Young and old wildtype mice, treated with PHP.*GFAP*-IL2 (or PHP.*GFAP*-GFP control vector) were assessed two months post-treatment by single cell sequencing using 10x single-cell transcriptomics. UMAP feature expression plots for markers characteristic of non-target populations present in the dataset: **A)** reticulocytes, **B)** neutrophils, **C)** perivascular macrophages, **D)** Choroid plexus epithelial cells, **E)** neurons, **F)** olfactory ensheathing cells, **G)** olfactory neurons, **H)** interneurons, **J)** neuro stem and progenitor cells, **K)** ependymal cells, **L)** hypendymal cells, **M)** vascular endothelial cells, **N)** vascular leptomeningeal cells, **O)** pericytes and **P)** vascular smooth muscle cells. Color indicates expression level.

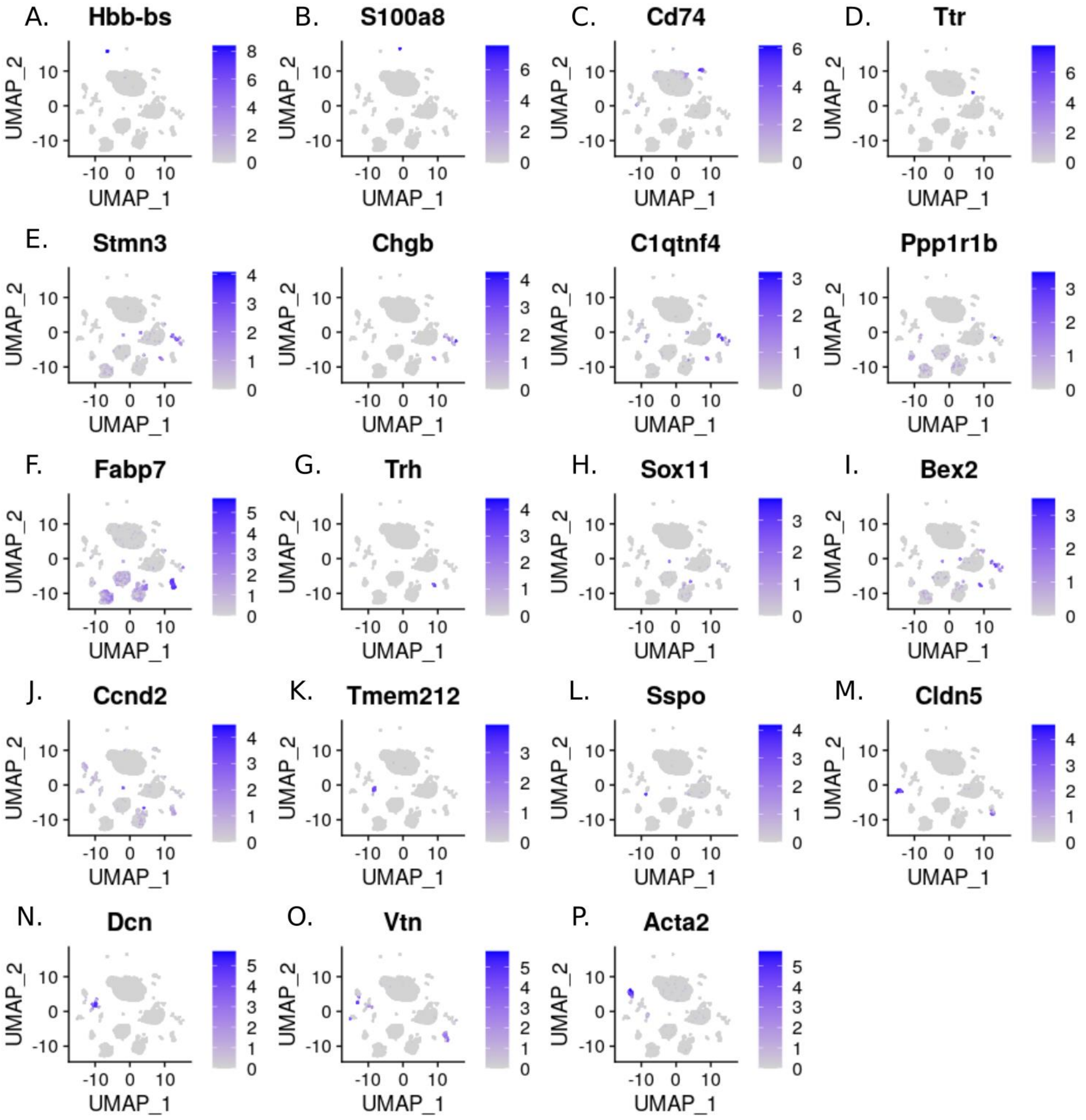

Appendix Figure S3. Expression of key homeostatic and activation markers within the astrocytic populations. Young and old wildtype mice, treated with PHP.*GFAP*-IL2 (or PHP.*GFAP*-GFP control vector) were assessed two months post-treatment sequencing using 10x single-cell transcriptomics to characterize isolated glial cells. Astrocytes were identified based on marker expression (**Figure S1**) and reclustered/reprojected via UMAP. **A)** UMAP feature expression plots for Bergmann glia, **B)** cerebellar astrocytes, **C)** non-telencephalon astrocytes, **D)** olfactory astrocytes, **E)** striatal astrocytes and **F)** telencephalon astrocytes. **G)** Astrocyte expression data was mapped onto the astrocyte expression data available at mousebrain.org, using the Seurat integration pipeline to verify identified populations. Color indicates expression level.

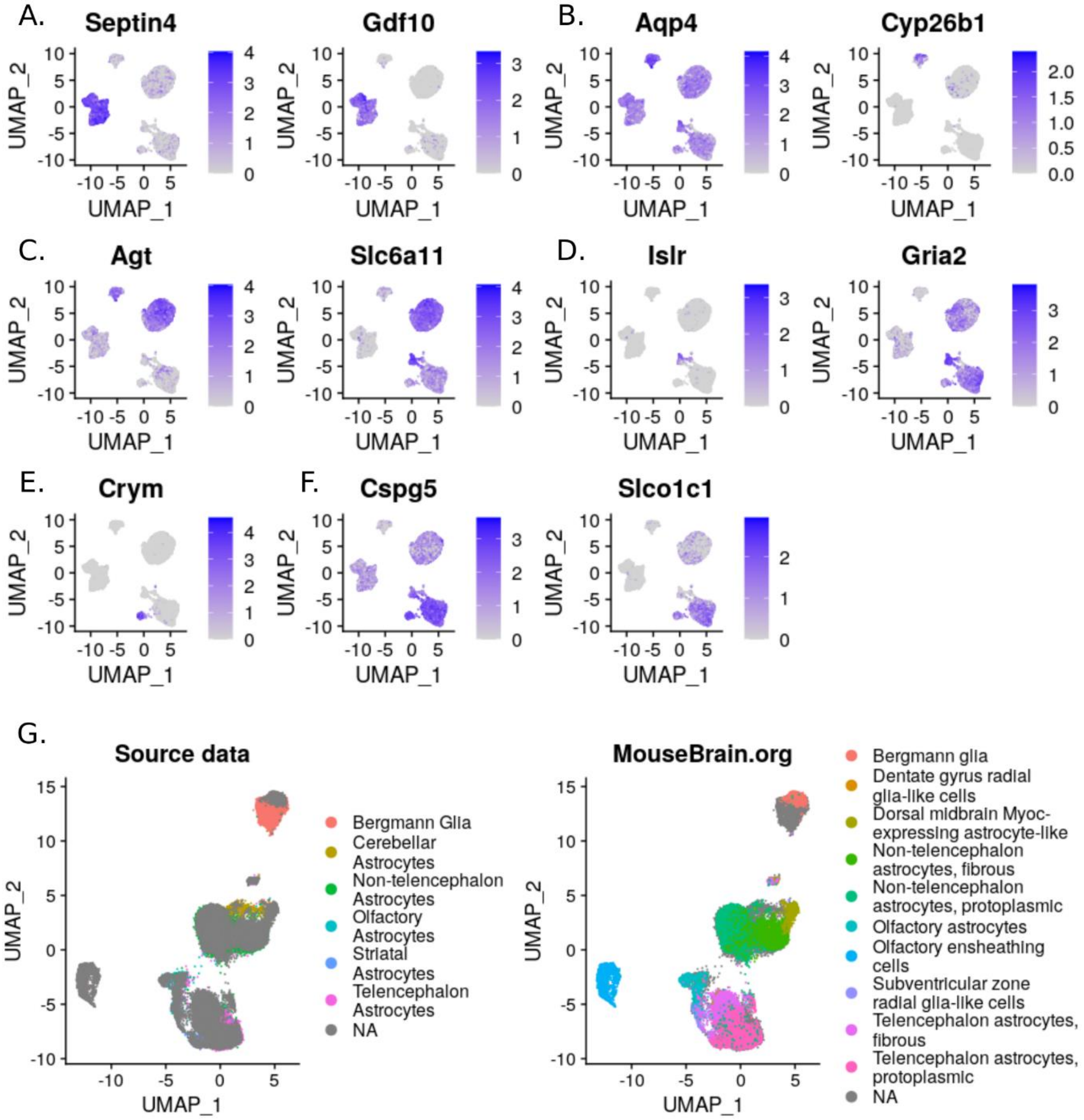

A.
Septin4
Gdf10
B.
Aqp4
Cyp26b1
C.
Agt
Slc6a11
D.
Islr
Gria2
E.
Crym
F.
Cspg5
Slco1c1
UMAP_1
UMAP_2
G.
Source data
Bergmann Glia
Cerebellar Astrocytes
Non-telencephalon Astrocytes
Olfactory Astrocytes
Striatal Astrocytes
Telencephalon Astrocytes
NA
MouseBrain.org
Bergmann glia
Dentate gyrus radial glia-like cells
Dorsal midbrain Myoc-expressing astrocyte-like
Non-telencephalon astrocytes, fibrous
Non-telencephalon astrocytes, protoplasmic
Olfactory astrocytes
Olfactory ensheathing cells
Subventricular zone radial glia-like cells
Telencephalon astrocytes, fibrous
Telencephalon astrocytes, protoplasmic
NA

Appendix Figure S4. Expression of key homeostatic and activation markers within the microglial populations. Young and old wildtype mice, treated with PHP.*GFAP*-IL2 (or PHP.*GFAP*-GFP control vector) were assessed two months post-treatment using 10x single-cell transcriptomics to characterize isolated glial cells. Microglia were identified based on marker expression (Keren-Shaul *et al* 2017) and reclustered/reprojected via UMAP. UMAP feature expression plots for **A)** homeostatic markers (*Cx3cr1*, *P2ry12*, *Tmem119, Cst3*, *Hexb*) and **B)** activation-associated markers (*Apoe*, *Lpl*, *Cst7, Axl*, *Itgax*, *Spp1, Ccl6*, *Csf1*). Color indicates expression level.

A.

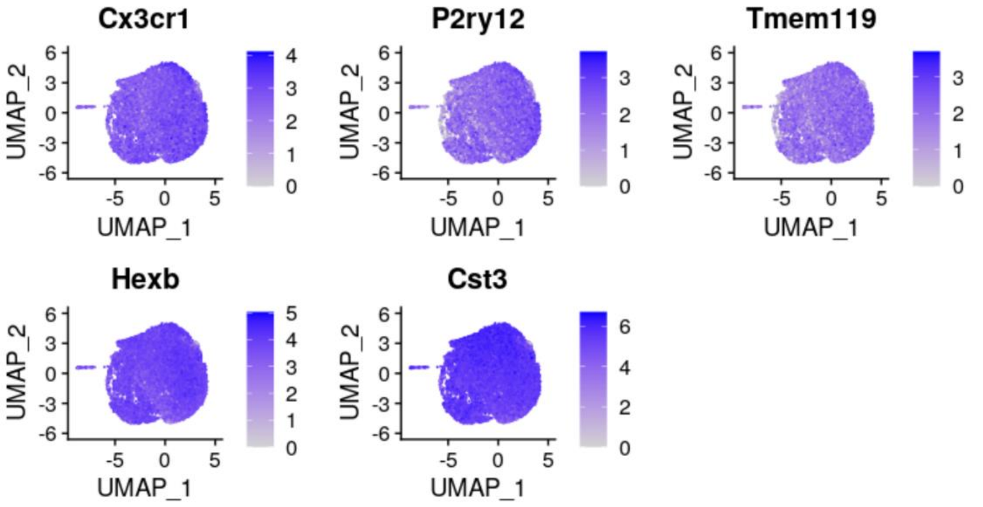
Cx3cr1
P2ry12
Tmem119
Hexb
Cst3
UMAP_1
UMAP_2

B.

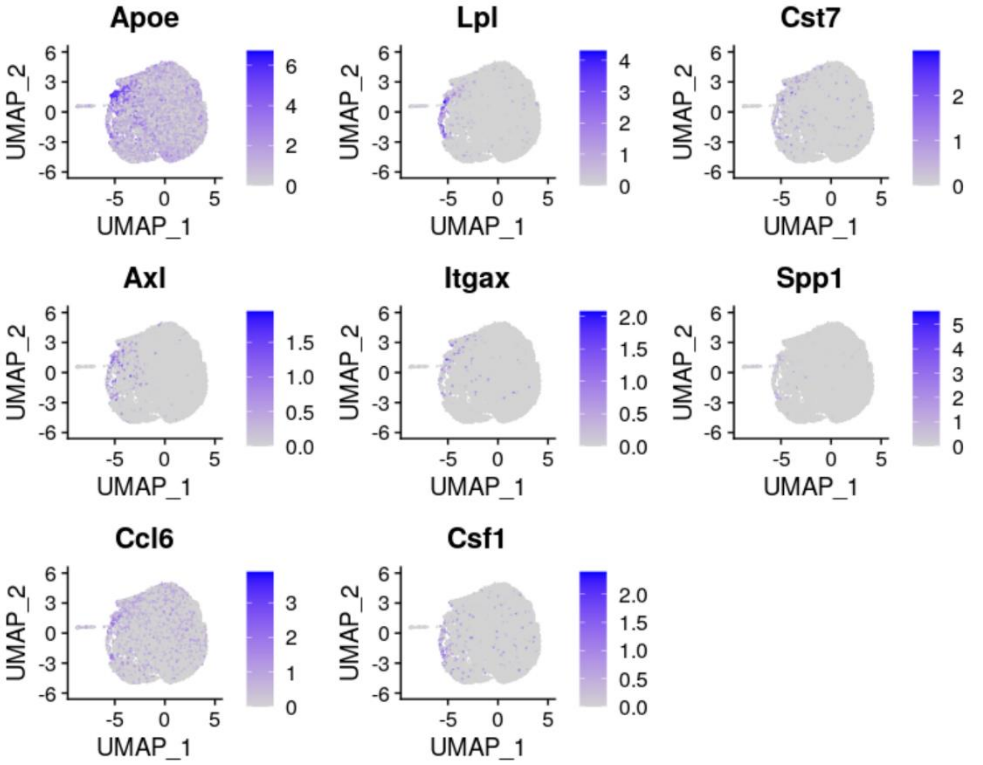
Apoe
Lpl
Cst7
Axl
Itgax
Spp1
Ccl6
Csf1
UMAP_1
UMAP_2

Appendix Figure S5. Expression of key homeostatic and activation markers within the oligodendrocyte and OPC populations. Young and old wildtype mice, treated with PHP.*GFAP*-IL2 (or PHP.*GFAP*-GFP control vector) were assessed two months post-treatment using 10x single-cell transcriptomics. Oligodendrocytes and oligodendrocyte precursor cells (OPCs) were identified based on marker expression and reclustered/reprojected into UMAP space. UMAP feature expression plots for key markers enriched in **A)** OLIGO1, **B)** OLIGO2, **C)** OLIGO3, **D)** OLIGO4, **E)** OLIGO5, and **F)** Oligodendrocyte precursor clusters. Color indicates expression level.

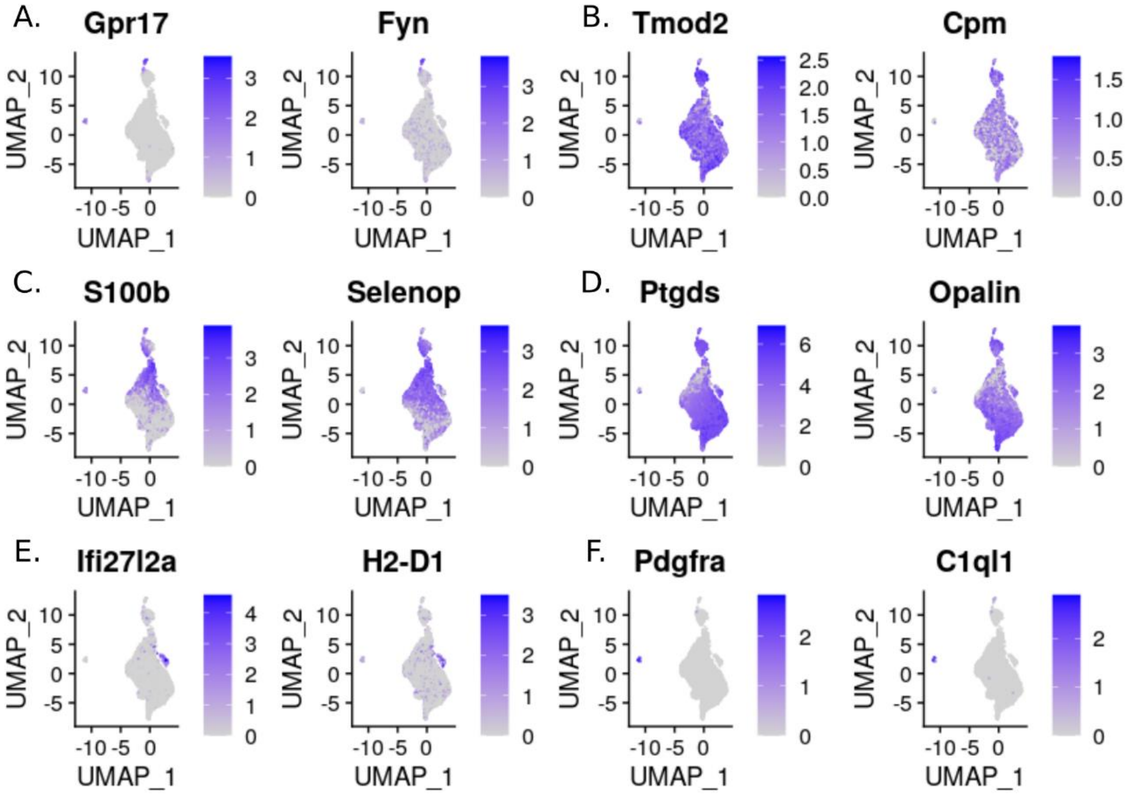

Supplement: Supplementary file 1 — Appendix [file EMMM-15-e16805-s006.pdf]
