# Supplementary material for: Molecular and cognitive signatures of ageing partially restored through synthetic delivery of IL2 to the brain
Source: EMBO Mol Med. 2023 Mar 28;15(5):e16805. doi: 10.15252/emmm.202216805 (PMC10165365; doi:10.15252/emmm.202216805)
Supplement: Supplementary file 2 — Expanded View Figures PDF [file EMMM-15-e16805-s002.pdf]

Expanded View Figures

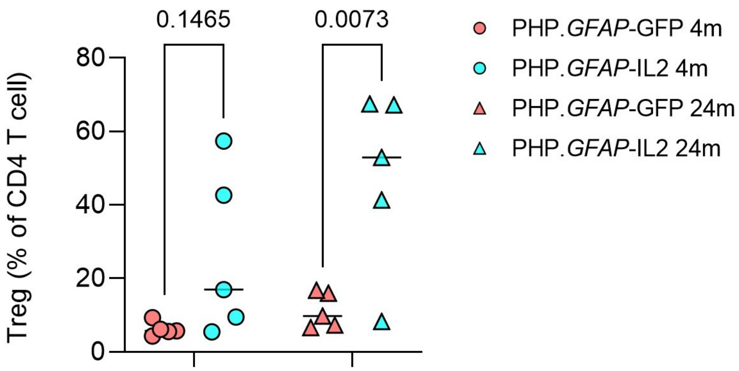

Figure EV1. PHP.*GFAP*-IL2 expands brain-resident regulatory T cells in aged mice.

Wild-type mice were treated with PHP.*GFAP*-GFP control vector or PHP.*GFAP*-IL2 at 2 months or 22 months of age, and were assessed for brain-resident Foxp3 + CD4+ Tregs by flow cytometry 2 months late (4 or 24 months of age). *N* = 5/group.

Data information: Mean and individual data points. 2-way ANOVA repeated measures with age and treatment as the main factors.

Source data are available online for this figure.

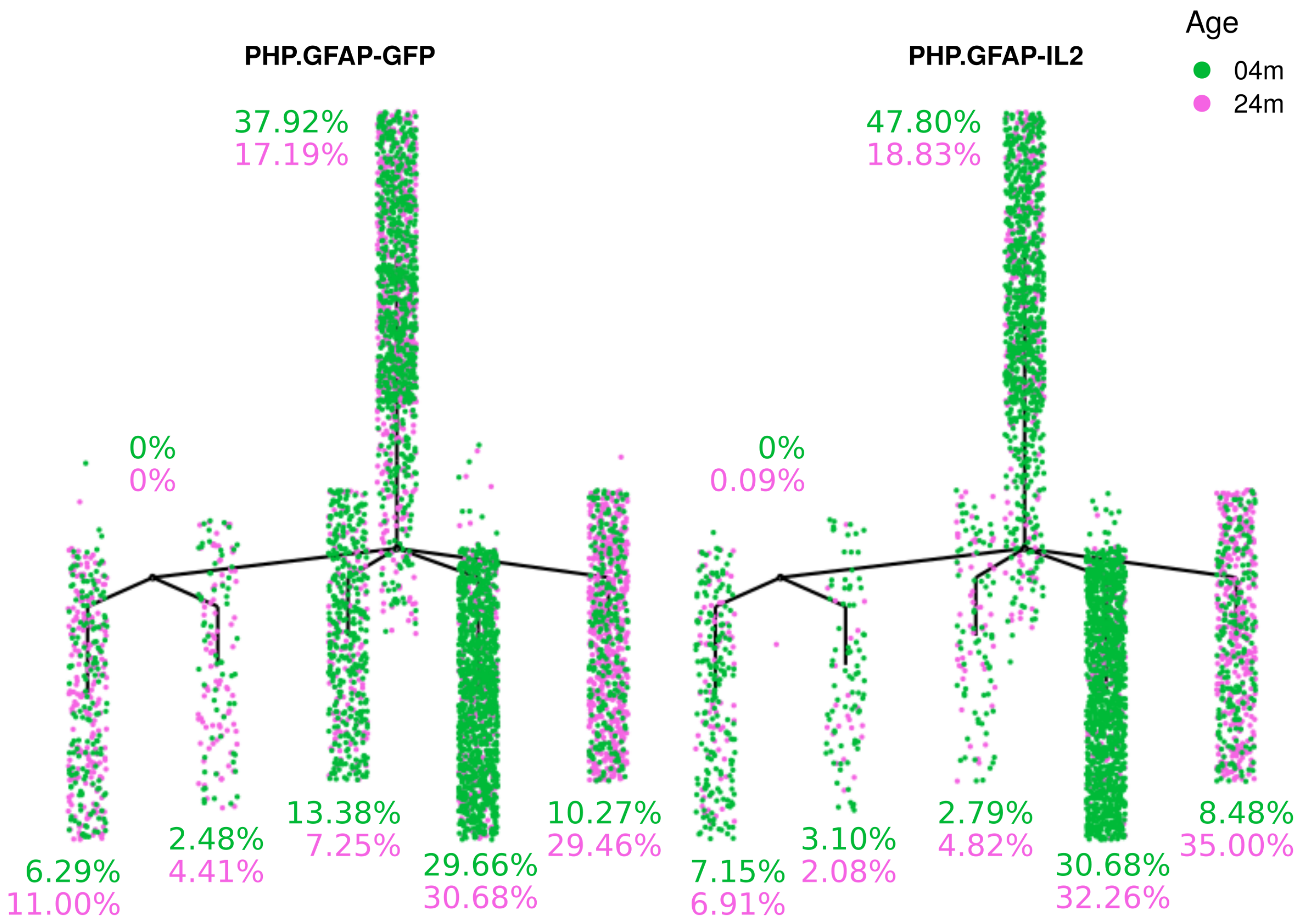

Figure EV2.

◀ **Figure EV2. Normal pseudotime cellular trajectory progression for oligodendrocytes following IL2-treatment.**

Wild-type mice were treated with PHP.*GFAP*-IL2 (or PHP.*GFAP*-GFP control vector) at 2 or 22 months of age (n = 3/group). Two-month post-treatment, the glial compartment was sorted from perfused mice and assessed using 10× single-cell transcriptomics. Oligodendrocytes and OPCs were reclustered and a pseudotime trajectory constructed (branching trajectory trees), using the DDRTree algorithm in Monocle v2. The tree was rooted at the branch with the highest proportion of young cells treated with PHP.*GFAP*-GFP. The trees were generated over the whole dataset and illustrated separately for PHP.*GFAP*-GFP and PHP.*GFAP*-IL2.

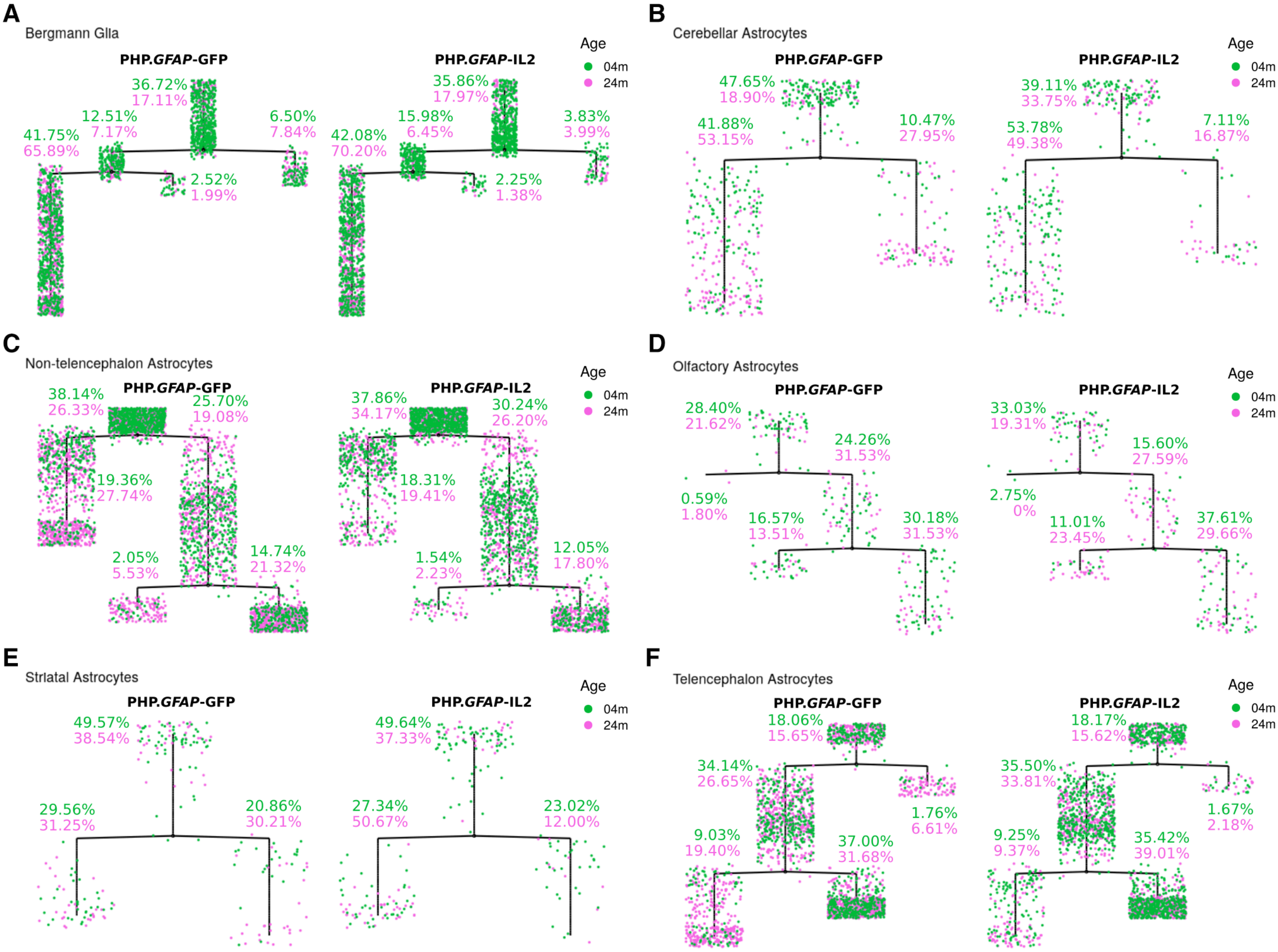

Figure EV3. Pseudotime trajectory trees for astrocytes during aging and following treatment with IL2.

Young and old wild-type mice, treated with PHP.*GFAP*-IL2 (or PHP.*GFAP*-GFP control vector) were assessed 2-month post-treatment by single-cell sequencing using 10x single-cell transcriptomics. Astrocytes were identified based on marker expression and reclustered/reprojected in UMAP space. Astrocyte subclusters, annotated based on key markers, were assessed for pseudotime trajectory. Pseudotime analysis generated branching trajectory trees of cells (based on the gene expression profile of each cell), using the DDRTree algorithm in Monocle v2. The tree was rooted at the branch with the highest proportion of young cells treated with PHP.*GFAP*-GFP.

A–F (A) The trees were generated over the whole data and illustrated separately for PHP.*GFAP*-GFP and PHP.*GFAP*-IL2 for Bergmann glia, (B) cerebellar astrocytes, (C) nontelencephalon astrocytes, (D) olfactory astrocytes, (E) striatal astrocytes and (F) telencephalon astrocytes.

 © 2023 The Authors

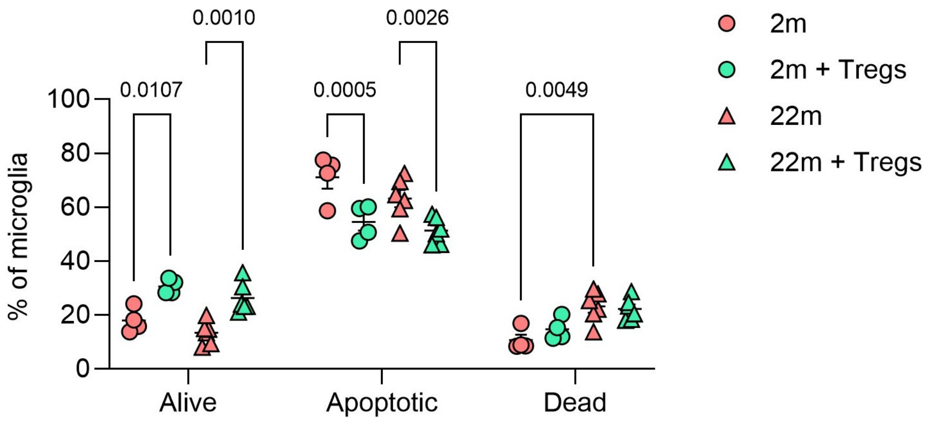

Figure EV4. *In vitro* effects of Treg co-culture on microglial survival.

Primary microglia isolated from adult or aged mice were sort-purified and plated for 2 days, prior to co-culturing with or without sort-purified Tregs, supplemented with 20 ng/ml IL2, for 6 days. At the end point, microglia were assessed for apoptosis using flow cytometry, using AnnV and PI.

Data information: Mean ± SEM of technical replicates; 2-way ANOVA with age and treatment as the main factors. Representative of experiment repeated twice.
Source data are available online for this figure.

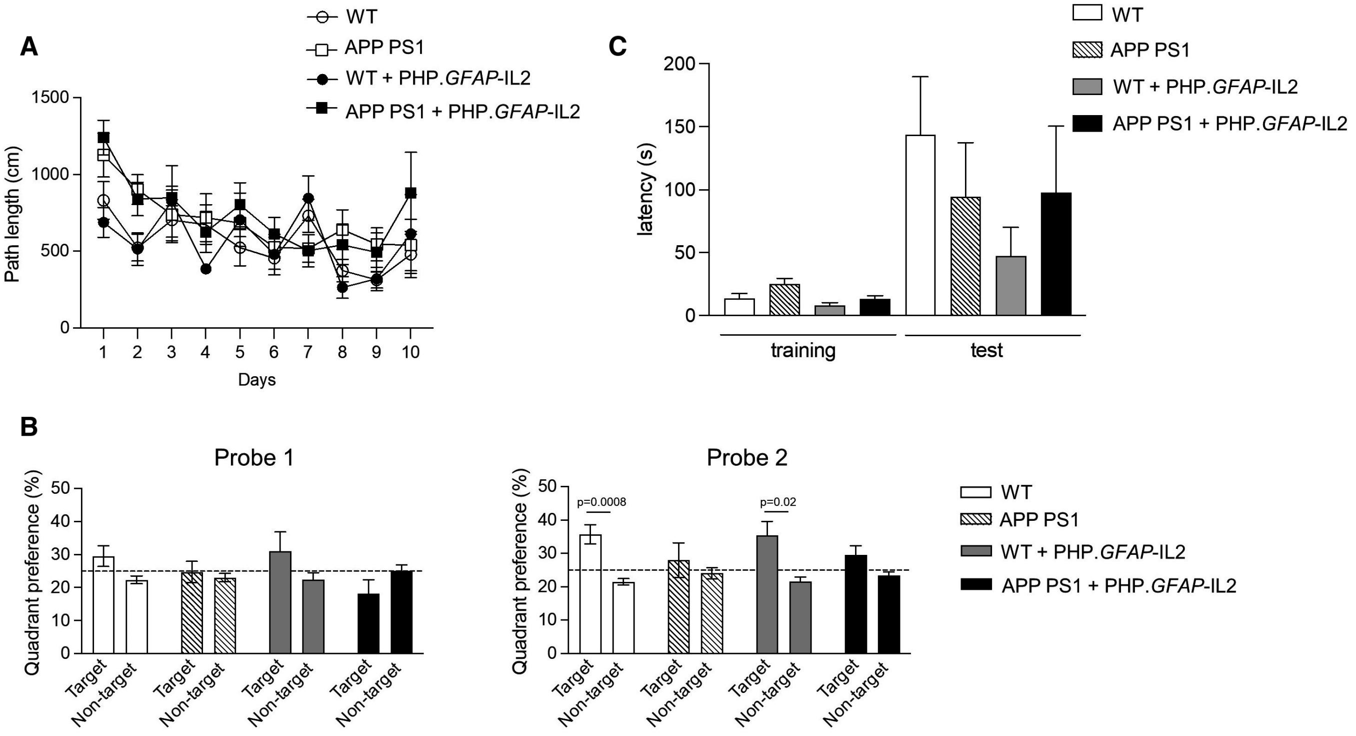

Figure EV5. Behavioral assessment of APP PS1 mice treated with PHP.*GFAP*-IL-2.

Female APP-PS1 and littermate controls were administered PHP.*GFAP*-IL2 (10^9 vg/mouse) i.v. at 2 months of age, and behavioral tests were performed at 10 months of age.
A Spatial learning in the Morris Water Maze. Path length to finding the hidden platform (*n* = 9, 7, 5, 7).
B Probe tests after 5 days, 10 days and after reversal learning (*n* = 9, 7, 5, 7). Dashed line represents random chance (25%) of quadrant preference.
C Performance in the passive avoidance test (*n* = 9, 7, 5, 7).

Data information: Mean ± SEM. (B and C) 2-way ANOVA with genotype and treatment as the main factors.
Source data are available online for this figure.

© 2023 The Authors
