# Supplementary material for: Molecular and cognitive signatures of ageing partially restored through synthetic delivery of IL2 to the brain
Source: EMBO Mol Med. 2023 Mar 28;15(5):e16805. doi: 10.15252/emmm.202216805 (PMC10165365; doi:10.15252/emmm.202216805)
Supplement: Supplementary file 11 [file EMMM-15-e16805-s011.pdf]

EMBO Molecular Medicine

Molecular and cognitive signatures of ageing partially restored through delivery of IL2 to the brain

Pierre Lemaitre, Samar Tareen, Emanuela Pasciuto, Loriana Mascali, Araks Martirosyan, Zsuzsanna Callaerts-Vegh, Suresh Poovathingal, James Dooley, Matthew Holt, Lidia Yshii, and Adrian Liston
DOI: 10.15252/emmm.202216805

Corresponding authors: Adrian Liston (adrian.liston@babraham.ac.uk) , Lidia Yshii (lidia.yshii@kuleuven.be), Matthew Holt (mholt@i3s.up.pt)

Review Timeline:

Submission Date:	28th Aug 22
Editorial Decision:	17th Oct 22
Revision Received:	19th Feb 23
Editorial Decision:	6th Mar 23
Revision Received:	8th Mar 23
Accepted:	10th Mar 23

Editor: Jingyi Hou

Transaction Report:

(Note: With the exception of the correction of typographical or spelling errors that could be a source of ambiguity, letters and reports are not edited. Depending on transfer agreements, referee reports obtained elsewhere may or may not be included in this compilation. Referee reports are anonymous unless the Referee chooses to sign their reports.)

17th Oct 2022

Dear Dr. Liston,

Thank you again for submitting your work to EMBO Molecular Medicine. We have now heard back from the two referees who agreed to evaluate your manuscript. As you will see from the reports below, the referees acknowledge the potential interest of the study. However, they raise a series of concerns, which we would ask you to address in a revision of the manuscript.

In particular, Referee #2's concerns about mechanisms must be carefully addressed, and this referee made constructive recommendations about specific experiments. We would not ask you to combine the current study with the BioRxiv paper referenced by Referee #1. However, considering that both referees mentioned that some of the findings had already been reported before, efforts should be made to better highlight the novel aspect of the study and place the current work in the existing literature.

During our pre-decision cross-commenting (in which the referees are given a chance to make additional comments, including on each other's reports), Referee #2 added, "if additional experiments are performed and a mechanism is included in the analyses, the paper could be considered acceptable for publication."

We would welcome the submission of a revised version within three months for further consideration. Please note that EMBO Molecular Medicine strongly supports a single round of revision. As acceptance or rejection of the manuscript will depend on another round of review, your responses should be as complete as possible.

EMBO Molecular Medicine has a "scooping protection" policy, whereby similar findings that are published by others during review or revision are not a criterion for rejection. Should you decide to submit a revised version, I do ask that you get in touch after three months if you have not completed it to update us on the status.

We are aware that many laboratories cannot function at full efficiency during the current COVID-19/SARS-CoV-2 pandemic and have therefore extended our "scooping protection policy" to cover the period required for a full revision to address the experimental issues. Please let me know should you need additional time, and also if you see a paper with related content published elsewhere.

Please read below for important editorial formatting and consult our author's guidelines for proper formatting of your revised article for EMBO Molecular Medicine.

I look forward to receiving your revised manuscript.

Use this link to login to the manuscript system and submit your revision: https://embomolmed.msubmit.net/cgi-bin/main.plex

Sincerely,
Jingyi

Jingyi Hou
Editor
EMBO Molecular Medicine

*****

***** Reviewer's comments *****

Referee #1 (Comments on Novelty/Model System for Author):

See below the comments to the authors

Referee #1 (Remarks for Author):

The manuscript is by an outstading team in the field. The described results are very thorough and solid.
Nevertheless, the results in the present manuscript are descriptive in nature, and simply extend the group's outstanding findings, which were extensively described in their previous publication, earlier this year.
Actually, it would make much more sense for the authors to combine the current results on ageing with their findings related to AD (https://www.biorxiv.org/content/10.1101/2022.03.11.483903v1) to make the manuscript more compelling, instead of publishing each set of results separately.
If this would be acceptable to the authors and to the journal, I think this would result in a very strong publication.

Referee #2 (Comments on Novelty/Model System for Author):

The neuroprotective role played by regulatory T cells (Treg) is well known. This includes but is not limited to the following.

Alzheimer's disease(https://pubmed.ncbi.nlm.nih.gov/?term=Regulatory+T+cells+neuroprotection+and+Alzheimer%27s+disease&sort=date); in Parkinson's disease (https://pubmed.ncbi.nlm.nih.gov/?term=Regulatory+T+cells +neuroprotection+and+Parkinson%27s+disease&sort=date); in stroke (https://pubmed.ncbi.nlm.nih.gov/?term=Regulatory+T+cells+neuroprotection+and+stroke&sort=date) and in amyotrophic lateral sclerosis (https://pubmed.ncbi.nlm.nih.gov/?term=Regulatory+T+cells+neuroprotection+and+ALS&sort=date).

Studies of the aging brain and Treg is of interest as is the mechanisms thereof and as result makes this paper of some interest. Nonetheless, the new findings need be made in the context of what has come before which in the current manuscript is more limited as IL-2 and IL-2 delivery in combating neurodegeneration is quite well established.

https://pubmed.ncbi.nlm.nih.gov/?term=Regulatory+T+cells+IL-2+and+neuroprotection&sort=date

a recent specific example is listed below....

Interleukin-2 expands neuroprotective regulatory T cells in Parkinson's disease. Milica Markovic et al. the journal NeuroImmune Pharmacology and Therapeutics. https://doi.org/10.1515/nipt-2022-0001

Referee #2 (Remarks for Author):

The current study describes the molecular and cognitive signatures of ageing in mice that is partially restored through IL2 treatments. The investigators tested brain-specific IL2 delivery for the prevention of neurological decline in aging mice. They identify molecular hallmarks of aging in the brain glial compartments, and identify partial restoration through IL2 treatment which includes defects in spatial learning without improvements in motor skills or arousal. The authors conclude that their results would identify immune modulation as a potential path to preserving cognitive.

This is an interesting study that expands on a large body of literature that has assessed the roles played by T cell subsets in a spectrum of neurodegenerative disease and in ageing. In particular a putative therapeutic role for IL2 and in particular agents that induce regulatory T cells in neuroprotection has been described in in vitro, ex vivo models or in early phase clinical tests.

The data is generally well presented but the dissections of links to microglia, astrocyte and oligodendrocyte processes are not clear from the data sets offered. In particular the critical role of microglia Functional decline of microglia which is arguably progressive over a lifespan with associated oxidative DNA damage and aggregates and protein-lipid modifications linked to neuroinflammation is not readily seen modified by the IL2 treatments offered. Figures 4 and 5 would benefit from attention in adding modeling of laboratory in vitro studies that would recapitulate the principal findings by exploring microglial activities during cell to cell interactions with IL2 enhanced T cells or subsets thereof to affirm the study's conclusions. Alternatively adoptive transfer serial increases IL2 T cells in the aging animal would be affirmative.

Referee #1

The manuscript is by an outstanding team in the field. The described results are very thorough and solid.
Nevertheless, the results in the present manuscript are descriptive in nature, and simply extend the group's outstanding findings, which were extensively described in their previous publication, earlier this year.
Actually, it would make much more sense for the authors to combine the current results on ageing with their findings related to AD (https://www.biorxiv.org/content/10.1101/2022.03.11.483903v1) to make the manuscript more compelling, instead of publishing each set of results separately.
If this would be acceptable to the authors and to the journal, I think this would result in a very strong publication.

We thank the reviewer for the positive evaluation of our past and present work. We also agree with the reviewer that the current work extends our Nature Immunology 2022 study, which demonstrated that brain Tregs were protective in neuroinflammatory models of traumatic brain injury, stroke and multiple sclerosis. We believe, however, that it is important to extend these results from neuroinflammation to neurodegeneration, as it is not a given that therapeutics with benefit in one set of conditions will necessarily have a benefit in another set of conditions. In particular, the inflammatory component of ageing is only just being explored, and as far as we know ours is the first study to look at the role of brain IL2/Tregs in natural ageing.

This point is made quite strongly by the example that the reviewer alludes to, our BioRxiv paper on Alzheimer's Disease models. While IL2/Tregs are strongly protective in neuroinflammatory diseases such as multiple sclerosis, the data in Alzheimer's Disease is much more equivocal, with only a small number of published studies, observing a range of outcomes from a beneficial effect, to little-or-no effect, to a detrimental effect. The degree to which natural ageing would fall on this spectrum between a neuroinflammatory disease like multiple sclerosis or an amyloid-driven disease like Alzheimer's, and thus the degree to which IL2/Treg treatment would be efficacious, was thus a genuinely open question before our study.

On the issue of merging the Yshii et al BioRxiv manuscript with the current manuscript, the editorial team has advised against, and we also believe that the merged manuscript would be unwieldy, for two reasons: 1) the Yshii et al BioRxiv manuscript is heavy on immune phenotyping data, which dramatically lengthens the manuscript, and 2) the Yshii et al BioRxiv paper uses a transgene for IL2 production in the brain while the current manuscript uses an AAV-based therapeutic. As there are key differences between the systems, this would make the comparison of the positive result observed with ageing to the negative result observed with Alzheimer's a bit like comparing apples and pears. We have, however, taken to heart the reviewer's advice to include Alzheimer's data in this paper. We therefore treated a new cohort of APP-PS1 transgenic mice with the same AAV-based system used in our ageing study (PHP.GFAP-IL2) and followed the mice out until amyloid-driven cognitive decline occurs. As in the Yshii et al BioRxiv paper, the brain IL2/Treg treatment gave no protection/improvement in this Alzheimer's model. We believe this is a better data set to be merged into this manuscript, which we have now done as the final figure of the paper. We also note that the separation of results observed in natural ageing vs amyloid-driven ageing emphases the importance of our study in extending beyond genetic/induced neuroinflammation to bona fide natural ageing.

Results:

“As a preliminary test to determine whether this protective effect against cognitive decline in healthy ageing could be extended to the amyloid-driven accelerated cognitive decline observed in Alzheimer’s Disease mouse models, we treated a cohort of pre-symptomatic APP-PS1 mice (Jankowsky, 2004) with PHP.*GFAP*-IL2 at 2 months of age. At 10 months of age, both treated and untreated APP-PS1 transgenic mice had similar levels of cognitive decline (**Fig EV5**). While not excluding potential efficacy at different doses or treatment regimes, these results suggest the IL2 treatment is less efficacious in amyloid-driven cognitive decline than in age-related cognitive decline.”

Expanded View Figure 5. Behavioral assessment of APP PS1 mice treated with PHP.*GFAP*-IL-2. Female APP-PS1 and littermate controls were administered PHP.*GFAP*-IL2 (10^9 vg/mouse) i.v. at 2 months of age, and behavioral tests were performed at 10 months of age. **A** Spatial learning in the Morris Water Maze. Path length to finding the hidden platform (n=9,7,5,7). **B** Probe tests after 5 days, 10 days and after reversal learning (n=9,7,5,7). **C** Performance in the passive avoidance test (n=9,7,5,7). Data information: Mean ± SEM. (B and C) 2-way ANOVA with genotype and treatment as the main factors.

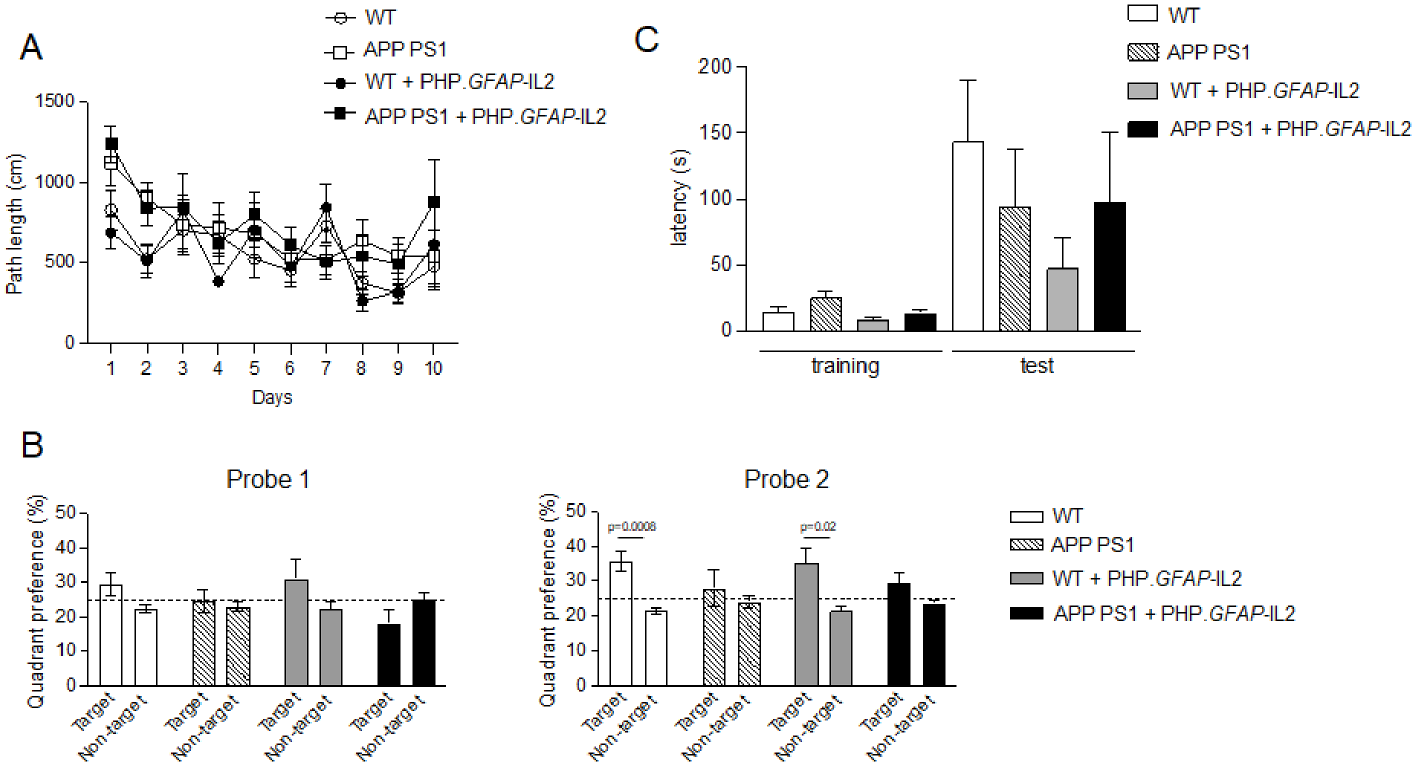

Discussion:

“A third limitation lies in the heterogeneity of causes for cognitive decline. Similar cognitive manifestations can develop from distinct molecular and cellular pathways impairing neurological networks. The degree to which the process is driven by inflammation is likely to vary from individual to individual, even within the aged population. Potential response to an IL2-based therapy will likely vary, as illustrated, for example, by the data shown here on protection from cognitive decline in a pure age-based model but not in a model driven by amyloid plaque formation.”

Referee #2

The current study describes the molecular and cognitive signatures of ageing in mice that is partially restored through IL2 treatments. The investigators tested brain-specific IL2 delivery for the prevention of neurological decline in aging mice. They identify molecular hallmarks of aging in the brain glial compartments, and identify partial restoration through IL2 treatment which includes defects in spatial learning without improvements in motor skills or arousal. The authors conclude that their results would identify immune modulation as a potential path to preserving cognitive.

This is an interesting study that expands on a large body of literature that has assessed the roles played by T cell subsets in a spectrum of neurodegenerative disease and in ageing. In particular a putative therapeutic role for IL2 and in particular agents that induce regulatory T cells in neuroprotection has been described in in vitro, ex vivo models or in early phase clinical tests.

> *We thank the reviewer for their positive comments. We would note that while work both by our group and others has elucidated aspects of Tregs function in neurodegenerative and neuroinflammatory disorders, it is by no means exhaustive, and in particular natural ageing has been largely neglected. This is detailed in more length below, with an accompanying expanded introduction on the topic.*

The data is generally well presented but the dissections of links to microglia, astrocyte and oligodendrocyte processes are not clear from the data sets offered. In particular the critical role of microglia Functional decline of microglia which is arguably progressive over a lifespan with associated oxidative DNA damage and aggregates and protein-lipid modifications linked to neuroinflammation is not readily seen modified by the IL2 treatments offered. Figures 4 and 5 would benefit from attention in adding modelling of laboratory in vitro studies that would recapitulate the principal findings by exploring microglial activities during cell to cell interactions with IL2 enhanced T cells or subsets thereof to affirm the study’s conclusions. Alternatively adoptive transfer serial increases IL2 T cells in the aging animal would be affirmative.

> *We certainly agree with the reviewer that while our study is an important step in studying Treg-glia interactions in ageing, it by no means closes the topic. While our study shows transcriptional rewiring of the glial component, with the functional outcome of improved cognition at the organism level, the full chain of cellular events that link brain IL2 treatment to improved cognitive performance remains to be determined. While these studies cannot all be performed within the current scope, and with ageing studies in particular it is important to consider the practical limitations of experimental slowness, we have taken the reviewer’s suggestion to start to develop in vitro models for microglia / Treg interaction. The vast majority of microglia in vitro assays are performed on fetal or neonatal microglia, as adult microglia die in culture. Microglia from aged mice performed even worse, with 90% of cells apoptotic or dead after 6 days of culture. This makes many of the in vitro assays that can be performed on fetal/neonatal microglia impossible to run for aged microglia. Surprisingly, however, this high rate of apoptosis was partially rescued by the provision of Tregs to the culture. This suggests that the fragility of adult/aged microglia in culture is in part due to missing interactions from Tregs. While it is extremely difficult to extrapolate between in vitro and in vivo systems (e.g., the apoptosis rate in vitro is vastly higher than that observed in vivo), this result is consistent with microglial protection from senescence, as even a minor protection from apoptosis in vivo would,*

over time, slow the cellular ageing process by reducing turnover. We believe that, while preliminary, this in vitro model provides an important tool for future investigations.

On the topic of using serial adoptive transfer of IL2 and Tregs into ageing animals, this is, unfortunately, beyond the scope of the current study, and would require an extensive new set of ageing studies to be set-up. While it would be of interest to compare different delivery approaches for IL2, we believe that the current approach (with a single treatment of an off-the-shelf viral vector) would be more practical in the aged patient context than serial adoptive cell therapy transfers.

We have added the new in vitro data to the paper, and expanded the discussion on the topic of mechanism:

Results:

"To develop an in vitro model for the impact of IL2 and Tregs on glial cells, we used microglial cultures. While fetal microglia survive well in culture, microglia from adult mice have poor survival in vitro. Microglia from aged mice had higher in vitro death rates than those purified from adult mice (**Fig EV4**). The provision of IL2 and Tregs to the culture media increased microglia survival and reduced apoptosis rates (**Fig EV4**). While care must be taken in extrapolation from in vitro to in vivo systems, as rate-limiting factors can vary in these contexts, these results provide a potential explanation for the molecular protection afforded by local IL2 production in vivo. Prevention of the aged/senescence transcriptional signature associated with local Treg expansion is consistent with long-term (minor) reductions in apoptosis rate and cellular turnover."

Expanded View Figure 4. In vitro effects of Treg co-culture on microglial survival. Primary microglia isolated from adult or aged mice were sort-purified and plated for two days, prior to co-culturing with or without sort-purified Tregs, supplemented with 20 ng/ml IL2, for 6 days. At the end point, microglia were assessed for apoptosis using flow cytometry, using AnnV and PI. Data information: Mean ± SEM of technical replicates; 2-way ANOVA with age and treatment as the main factors. Representative of experiment repeated twice.

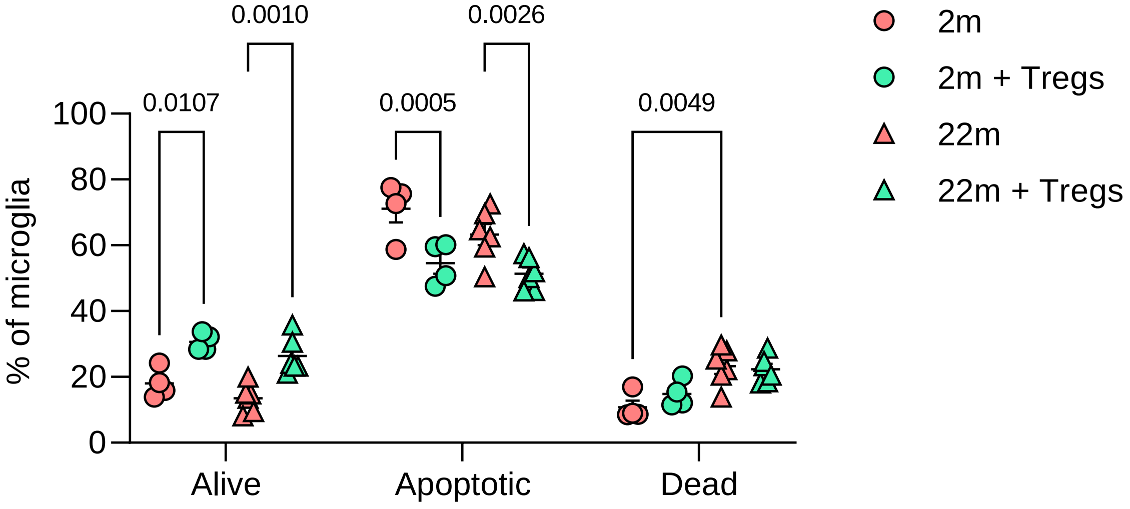

Discussion:

"Brain ageing is a multifaceted phenomenon, encompassing structural, functional and microenvironmental changes that impact on both the neuronal and glia compartments. However, the causative drivers of deleterious cellular changes are

difficult to untangle, as alterations such as loss of neuronal circuits and plasticity could be primarily driven by cell intrinsic neuronal mechanisms of ageing, or alternatively be derived from the impact of ageing glial support. The role of inflammation in this process is likely to be a primary driver of at least some aspects of the ageing process, based on correction experiments. For example, defects in learning and memory capacity in the contextual fear conditioning and radial arm water maze assays can be partially corrected in 18 months-old mice through the repeated injection of plasma from young mice {Villeda, 2014 #80}. Likewise, heterochronic parabiosis, joining the circulatory system of a 21 months-old mouse to a 2 months-old mouse, improved the performance of old mice in olfactory sensitivity assays (Katsimpardi, 2014). Thus, while a neuronal cell intrinsic ageing effect is likely, an emergent feature of cognitive decline must at least partly depend on environmental factors, such as a build-up of toxic or inflammatory products. T cells are a key potential player in the development of this neuroageing environment. T cells can directly produce, or stimulate the production of, key inflammatory mediators, including cytokines, ROS and autoantibodies, a process that can directly contribute to emergent features such as the constraint of the neuronal stem cell niche (Dulken, 2019). In particular, IFNγ production has emerged as a potential mediator of age-related decline, with activation of microglia and astrocytes leading to neuronal damage (Liston, 2023). The identification of T cells as a potential culprit opens the possibility of Tregs as a therapeutic. Tregs have direct anti-inflammatory properties, preventing excessively exuberant responses from conventional T cells. The protective effects observed here could potentially be driven through direct countering of T cell-mediated inflammation, although this modality of control would be restricted due to low densities of both cell types within the brain (Pasciuto, 2020). In addition, Tregs can produce pro-repair orientated cytokines, such as amphiregulin and osteopontin (Ito, 2019; Shi, 2021), which are neuroprotective following injuries such as stroke. As Treg numbers in the brain are low, it is expected that their influence will be mediated, at least in part, through the reprogramming of glial cells. In the peripheral context, Tregs are capable of reprogramming monocytes into the more pro-repair anti-inflammatory profile (Tiemessen, 2007). In the brain, an analogous program may be imparted onto microglia (Zhou, 2017), and our results here indicate potential for Tregs to slow cellular turnover of microglia, which may delay senescence. Microglial polarization can, in turn, impact astrocyte polarization (Li, 2020). Tregs can also stimulate OPC maturation into oligodendrocytes, promoting myelination, in a pro-repair state (Dombrowski, 2017). When considering the efficacy of PHP.GFAP-IL2 in mitigating ageing effects, the most parsimonious explanation would be a primary effect via the local expansion of Tregs. When used in the context of traumatic brain injury, the beneficial effects of PHP.GFAP-IL2 were only observed in the presence of an adaptive immune system, suggesting that the levels of IL2 achieved (~2pg/ml) are too low to trigger the activation of the lower affinity receptor expressed in non-Treg lineages (Yshii, 2022). While it would be attractive to speculate on the role of individual downstream mediators, the complexity and interdependencies of ageing make a multifactorial function more likely, which integrate pro-repair, anti-inflammatory and glial reprogramming functions. The in vitro system developed here may allow future dissection of some of these mechanisms.”

The neuroprotective role played by regulatory T cells (Treg) is well known. This includes but is not limited to the following.

Alzheimer's disease; in Parkinson's disease; in stroke and in amyotrophic lateral sclerosis [links]. Studies of the aging brain and Treg is of interest as is the mechanisms thereof and as result makes this paper of some interest. Nonetheless, the new findings need be made in the context of what has come before which in the current manuscript is more limited as IL-2 and

IL-2 delivery in combating neurodegeneration is quite well established.

a recent specific example is listed below....

Interleukin-2 expands neuroprotective regulatory T cells in Parkinson's disease. Milica Markovic et al. the journal NeuroImmune Pharmacology and Therapeutics.

> *We certainly agree with the reviewer that Tregs have been studied in multiple contexts of neuroinflammation and, to a lesser degree, in neurodegeneration. This was briefly, but insufficiently, described in the original introduction. However, the experiments to date are limited, and almost exclusively use methodologies that cannot distinguish between systemic and brain-resident based responses (e.g., most experiments use intravenous injection of Tregs or IL2, where the dominant effects may be mediated by shutting down peripheral inflammatory reactions or trafficking).*
>
> *We also believe that it is important to note that while genetic or induced models of neuroinflammation and neurodegeneration have been studied to some extent, as far as we know, ours is the first study to look at the role of brain IL2/Tregs in natural ageing. This is important for two key reasons: first, treatment of ageing will require therapeutic modalities different to those used in disease, and our strategy is more suitable for clinical translation than many of the approaches used in other pre-clinical studies. Second, natural ageing is a distinct biological process to disease-model approaches. For example, amyloid deposition is far more prominent in Alzheimer's Disease than it is in mild cognitive decline, and CD8 T cell infiltration occurs to a much greater degree in multiple sclerosis than in mild cognitive decline. It is, therefore, necessary to test age-related cognitive decline as a distinct entity, which may or may not respond to the same treatment approaches. On this latter point, the data added in reply to reviewer 1 is relevant – the IL2 delivery system that works here for age-related cognitive decline does not work in the amyloid-driven APP-PS1 model of Alzheimer's Disease.*
>
> *An extensive new section has been added to the manuscript, describing data obtained with the APP-PS1 model and appropriate changes have been made to the Introduction, Results and Discussion.*
>
> > Introduction:
> >
> > "Tregs have been proposed to have a protective function in a variety of neuroinflammatory and/or neurodegenerative diseases. Proposed therapeutic strategies to harness Tregs are typically based on either direct delivery of Tregs, through cell-based approaches, or the provision of biologics to enhance Tregs, often IL2, the key survival factor (Pierson *et al*, 2013). The results have, however, been inconsistent across models and with different modalities of treatment. For example, in Alzheimer's disease, direct cell therapy with Tregs (Baek *et al*, 2016; Faridar *et al*, 2022; Yang *et al*, 2022) has given beneficial effects, although other studies have shown the reverse (Baruch *et al*, 2015; Yang *et al*, 2020), while treatment with IL2 has given conflicting results, depending on the AD model, ranging from protective (Alves *et al*, 2017; Baek *et al.*, 2016) to minor (Dansokho *et al*, 2016) or no effect (Yshii *et al*, 2022a). In Parkinson's models, a number of different Treg-based treatments have been beneficial, including direct cell transfer (Huang *et al*, 2020; Markovic *et al*, 2022; Reynolds *et al*, 2007), using bee venom phospholipase A2 to induce Tregs, superagonist anti-CD28 antibodies to expand them (Badr *et al*, 2022) or vasoactive intestinal peptide receptor-2 (VIPR2) peptide agonist to enhance activity (Mosley *et al*, 2019). In stroke models, beneficial effects have been shown with direct Treg cell therapy (Ito *et al*, 2019) (although this only works in Rag-deficient

mice) or via the use of IL33 (Liu *et al*, 2020) or IL2 to expansion the Treg population (Dong *et al*, 2021; Shi *et al*, 2021; Yshii *et al*, 2022b; Zhang *et al*, 2018). Finally, in ALS models, both Treg cell therapy (Beers *et al*, 2011) and IL2 treatment (Sheean *et al*, 2018) have shown beneficial results. While critical in supporting a key role for Tregs in preventing pathology in neurodegenerative diseases, many of these studies use systems that do not distinguish between direct neuroprotection via brain-resident Tregs or indirect protection through control of peripheral inflammatory reaction by systemic Tregs.

Despite the growing recognition of a protective role of IL2 and Tregs in a range of neuroinflammatory and neurodegenerative processes, the pathological processes occurring in age are distinct from those in disease, and thus the potential of these cells to protect the ageing brain remain unknown."

Results:

"As a preliminary test to determine whether this protective effect against cognitive decline in healthy ageing could be extended to the amyloid-driven accelerated cognitive decline observed in Alzheimer's Disease mouse models, we treated a cohort of pre-symptomatic APP-PS1 mice (Jankowsky, 2004) with PHP.*GFAP*-IL2 at 2 months of age. At 10 months of age, both treated and untreated APP-PS1 transgenic mice had similar levels of cognitive decline (**Fig EV5**). While not excluding potential efficacy at different doses or treatment regimes, these results suggest the IL2 treatment is less efficacious in amyloid-driven cognitive decline than in age-related cognitive decline."

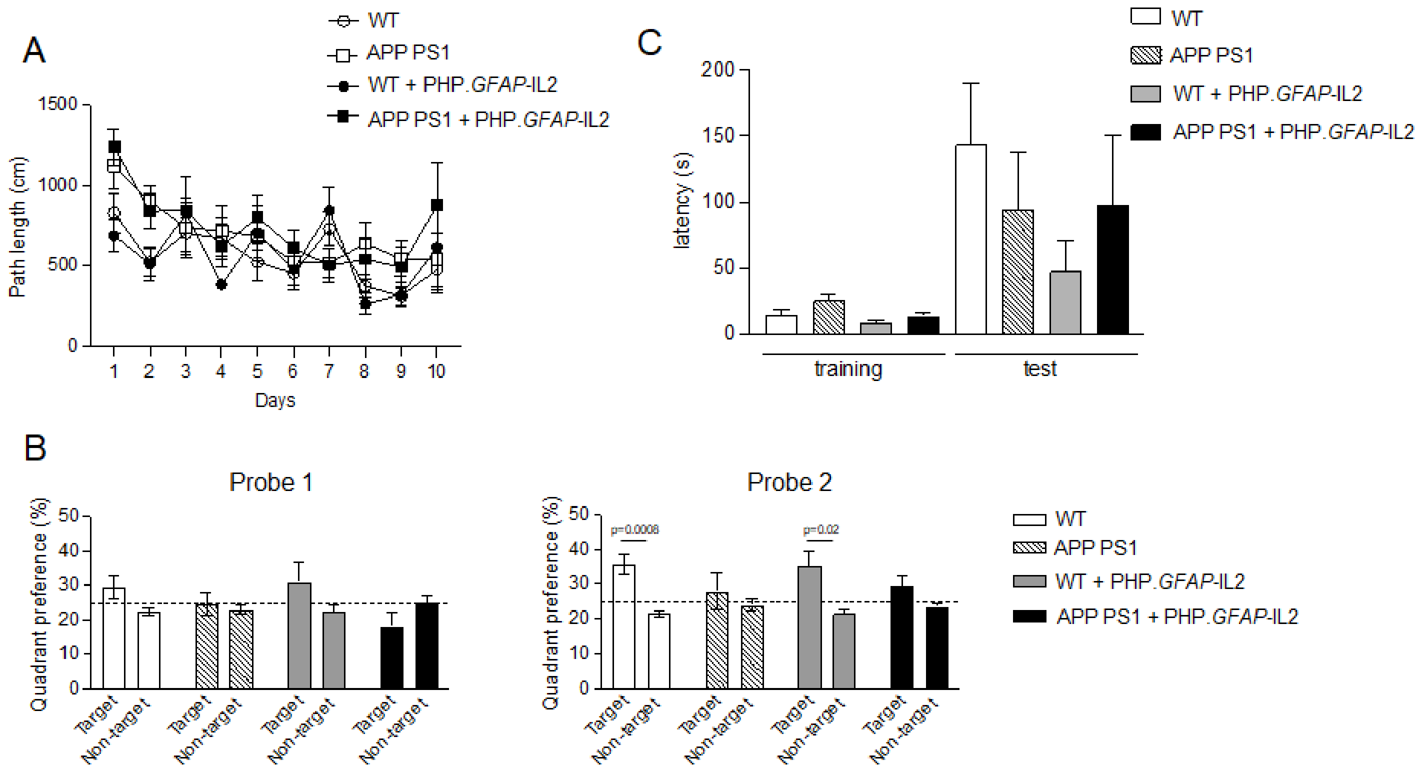

Expanded View Figure 5. Behavioral assessment of APP PS1 mice treated with PHP.*GFAP*-IL-2. Female APP-PS1 and littermate controls were administered PHP.*GFAP*-IL2 (10^9 vg/mouse) i.v. at 2 months of age, and behavioral tests were performed at 10 months of age. **A** Spatial learning in the Morris Water Maze. Path

length to finding the hidden platform (n=9,7,5,7). **B** Probe tests after 5 days, 10 days and after reversal learning (n=9,7,5,7). **C** Performance in the passive avoidance test (n=9,7,5,7). Data information: Mean ± SEM. (B and C) 2-way ANOVA with genotype and treatment as the main factors.

Discussion:

“A third limitation lies in the heterogeneity of causes for cognitive decline. Similar cognitive manifestations can develop from distinct molecular and cellular pathways impairing neurological networks. The degree to which the process is driven by inflammation is likely to vary from individual to individual, even within the aged population. Potential response to an IL2-based therapy will likely vary, as illustrated, for example, by the data shown here on protection from cognitive decline in a pure age-based model, but not in a model driven by amyloid plaque formation.”

6th Mar 2023

Dear Prof. Liston,

Thank you for sending us your revised manuscript. We have now received the comments from one of the two referees who were asked to re-review your manuscript. As you will see below, the referee is satisfied with the modifications and thinks the study is now suitable for publication.

Before we can formally accept your manuscript, we would ask you to address the following editorial-level issues:

1. Remove figures from the main manuscript file.

2. Figure callouts are missing for panels A, B, C of Figures 5 and 6 and panels A, B, C of Fig EV3 and 5. Please fix them.

3. Rename "Conflict of interest" to "Disclosure statement and competing interests".

4. Please ensure the funding information entered in the online submission system is consistent with that in the manuscript file.

5. Author checklist: Enter all three corresponding authors.

6. Rename Appendix Resources 1 and 2 to "Dataset EV1" and "EV2"; add the legends to the xlsx. files in a separate tab. Update the callouts.

7. Appendix

- Remove Appendix Resources 1 and 2 and source data; figures and their legends should stay.
- Add page numbers to the Table of Content.

8. Source data: Please complete the source data checklist.

9. Please provide a striking image or visual abstract to illustrate your article as a PNG file 550 px wide x 300-600 px high.

10. I have slightly modified the synopsis text (see attached). Please let me know if it is fine or if you would like to introduce further modifications.

11. Our data editors have seen the manuscript, and they have made some comments and suggestions that need to be addressed (see attached). Please send back a revised version (in track change mode), as we will need to go through the changes.

12. As part of the EMBO Publications transparent editorial process initiative (see our Editorial at http://embomolmed.embopress.org/content/2/9/329), EMBO Molecular Medicine will publish online a Review Process File (RPF) to accompany accepted manuscripts.

In the event of acceptance, this file will be published in conjunction with your paper and will include the anonymous referee reports, your point-by-point response, and all pertinent correspondence relating to the manuscript. Let us know if you disagree with this and if you want to remove or keep any figures from it prior to publication.

Please note that the Authors checklist will be published at the end of the RPF.

Please submit your revised manuscript within two weeks. I look forward to seeing a revised form of your manuscript as soon as possible.

Kind regards,
Jingyi

Jingyi Hou
Editor
EMBO Molecular Medicine

***** Reviewer's comments *****

Referee #2 (Comments on Novelty/Model System for Author):

IL-2 is well known to affect the pathobiology of neurodegenerative disorder. An example is shown below (https://pubmed.ncbi.nlm.nih.gov/28003243/). This is also true for autoimmune diseases and product development for clinical use is already advancing.https://www.businesswire.com/news/home/20230215005211/en/Coya-Therapeutics-Inc.-Announces-Expansion-of-Exclusive-Worldwide-Licensing-Agreement-with-ARScience-Biotherapeutics-Inc.-for-Development-and-Commercialization-of-COYA-301-Coya's-Low-Dose-Interleukin-2-IL-2-Product-Candidate. Thus while the technical quality is high the impact and novelty is medium.

Referee #2 (Remarks for Author):

The responses to my prior critique are adequate.

The authors addressed the remaining editorial issues.

10th Mar 2023

Dear Prof. Liston,

I am pleased to inform you that your manuscript is accepted for publication and is now being sent to our publisher to be included in the next available issue of EMBO Molecular Medicine.

We would like to remind you that as part of the EMBO Publications transparent editorial process initiative, EMBO Molecular Medicine will publish a Review Process File online to accompany accepted manuscripts. If you do NOT want the file to be published or would like to exclude figures, please immediately inform the editorial office via e-mail.

Please read below for additional IMPORTANT information regarding your article, its publication and the production process.

Congratulations on your interesting work,

Jingyi

Jingyi Hou
Editor
EMBO Molecular Medicine

Follow us on Twitter @EmboMolMed
Sign up for eTOCs at embopress.org/alertsfeeds

*** *** *** IMPORTANT INFORMATION *** *** ***

SPEED OF PUBLICATION
The journal aims for rapid publication of papers, using using the advance online publication "Early View" to expedite the process: A properly copy-edited and formatted version will be published as "Early View" after the proofs have been corrected. Please help the Editors and publisher avoid delays by providing e-mail address(es), telephone and fax numbers at which author(s) can be contacted.

Should you be planning a Press Release on your article, please get in contact with embomolmed@wiley.com as early as possible, in order to coordinate publication and release dates.

LICENSE AND PAYMENT:

All articles published in EMBO Molecular Medicine are fully open access: immediately and freely available to read, download and share.

EMBO Molecular Medicine charges an article processing charge (APC) to cover the publication costs. You, as the corresponding author for this manuscript, should have already received a quote with the article processing fee separately. Please let us know in case this quote has not been received.

Once your article is at Wiley for editorial production you will receive an email from Wiley's Author Services system, which will ask you to log in and will present you with the publication license form for completion. Within the same system the publication fee can be paid by credit card, an invoice, pro forma invoice or purchase order can be requested.

Payment of the publication charge and the signed Open Access Agreement form must be received before the article can be published online.

PROOFS

You will receive the proofs by e-mail approximately 2 weeks after all relevant files have been sent o our Production Office. Please return them within 48 hours and if there should be any problems, please contact the production office at embopressproduction@wiley.com.

Please inform us if there is likely to be any difficulty in reaching you at the above address at that time. Failure to meet our

deadlines may result in a delay of publication.

All further communications concerning your paper proofs should quote reference number EMM-2022-16805-V3 and be directed to the production office at embopressproduction@wiley.com.

Thank you,

Jingyi Hou
Editor
EMBO Molecular Medicine

EMBO Press Author Checklist

Corresponding Author Name: Adrian Liston, Matthew Holt, Lidia Yshii	
Journal Submitted to: EMBO Mol Med	
Manuscript Number: EMM-2022-16805	

USEFUL LINKS FOR COMPLETING THIS FORM
The EMBO Journal - Author Guidelines
EMBO Reports - Author Guidelines
Molecular Systems Biology - Author Guidelines
EMBO Molecular Medicine - Author Guidelines

Reporting Checklist for Life Science Articles (updated January 2022)
This checklist is adapted from Materials Design Analysis Reporting (MDAR) Checklist for Authors. MDAR establishes a minimum set of requirements in transparent reporting in the life sciences (see Statement of Task: 10.31222/osf.io/9sm4x). Please follow the journal's guidelines in preparing your manuscript.
Please note that a copy of this checklist will be published alongside your article.

Abridged guidelines for figures
1. Data
The data shown in figures should satisfy the following conditions:
- the data were obtained and processed according to the field's best practice and are presented to reflect the results of the experiments in an accurate and unbiased manner.
- ideally, figure panels should include only measurements that are directly comparable to each other and obtained with the same assay.
- plots include clearly labeled error bars for independent experiments and sample sizes. Unless justified, error bars should not be shown for technical replicates.
- if n<5, the individual data points from each experiment should be plotted. Any statistical test employed should be justified.
- Source Data should be included to report the data underlying figures according to the guidelines set out in the authorship guidelines on Data Presentation.

2. Captions
Each figure caption should contain the following information, for each panel where they are relevant:
- a specification of the experimental system investigated (eg cell line, species name).
- the assay(s) and method(s) used to carry out the reported observations and measurements.
- an explicit mention of the biological and chemical entity(ies) that are being measured.
- an explicit mention of the biological and chemical entity(ies) that are altered/varied/perturbed in a controlled manner.
- the exact sample size (n) for each experimental group/condition, given as a number, not a range;
- a description of the sample collection allowing the reader to understand whether the samples represent technical or biological replicates (including how many animals, litters, cultures, etc.).
- a statement of how many times the experiment shown was independently replicated in the laboratory.
- definitions of statistical methods and measures:
  - common tests, such as t-test (please specify whether paired vs. unpaired), simple χ2 tests, Wilcoxon and Mann-Whitney tests, can be unambiguously identified by name only, but more complex techniques should be described in the methods section;
  - are tests one-sided or two-sided?
  - are there adjustments for multiple comparisons?
  - exact statistical test results, e.g., P values = x but not P values < x;
  - definition of 'center values' as median or average;
  - definition of error bars as s.d. or s.e.m.

Please complete ALL of the questions below.
Select "Not Applicable" only when the requested information is not relevant for your study.

Materials

Newly Created Materials	Information included in the manuscript?	In which section is the information available? (Reagents and Tools Table, Materials and Methods, Figures, Data Availability Section)
New materials and reagents need to be available; do any restrictions apply?	Not Applicable	

Antibodies	Information included in the manuscript?	In which section is the information available? (Reagents and Tools Table, Materials and Methods, Figures, Data Availability Section)
For **antibodies** provide the following information:<br>- Commercial antibodies: RRID (if possible) or supplier name, catalogue number and or/clone number<br>- Non-commercial: RRID or citation	Yes	Materials and Methods

DNA and RNA sequences	Information included in the manuscript?	In which section is the information available? (Reagents and Tools Table, Materials and Methods, Figures, Data Availability Section)
**Short novel DNA or RNA including primers, probes**: provide the sequences.	Not Applicable	

Cell materials	Information included in the manuscript?	In which section is the information available? (Reagents and Tools Table, Materials and Methods, Figures, Data Availability Section)
**Cell lines:** Provide species information, strain. Provide accession number in repository **OR** supplier name, catalog number, clone number, and/**OR** RRID.	Not Applicable	
**Primary cultures:** Provide species, strain, sex of origin, genetic modification status.	Yes	Material and Methods
Report if the cell lines were recently **authenticated** (e.g., by STR profiling) and tested for mycoplasma contamination.	Not Applicable	

Experimental animals	Information included in the manuscript?	In which section is the information available? (Reagents and Tools Table, Materials and Methods, Figures, Data Availability Section)
**Laboratory animals or Model organisms:** Provide species, strain, sex, age, genetic modification status. Provide accession number in repository **OR** supplier name, catalog number, clone number, **OR** RRID.	Yes	Materials and Methods
**Animal observed in or captured from the field:** Provide species, sex, and age where possible.	Not Applicable	
Please detail **housing and husbandry conditions**.	Yes	Materials and Methods

Plants and microbes	Information included in the manuscript?	In which section is the information available? (Reagents and Tools Table, Materials and Methods, Figures, Data Availability Section)
**Plants:** provide species and strain, ecotype and cultivar where relevant, unique accession number if available, and source (including location for collected wild specimens).	Not Applicable	
**Microbes:** provide species and strain, unique accession number if available, and source.	Not Applicable	

Human research participants	Information included in the manuscript?	In which section is the information available? (Reagents and Tools Table, Materials and Methods, Figures, Data Availability Section)
If collected and within the bounds of privacy constraints report on age, sex and gender or ethnicity for all study participants.	Not Applicable	

Core facilities	Information included in the manuscript?	In which section is the information available? (Reagents and Tools Table, Materials and Methods, Figures, Data Availability Section)
If your work benefited from core facilities, was their service mentioned in the acknowledgments section?	Yes	Acknowledgements

Design

Study protocol	Information included in the manuscript?	In which section is the information available? (Reagents and Tools Table, Materials and Methods, Figures, Data Availability Section)
If study protocol has been **pre-registered, provide DOI in the manuscript**. For clinical trials, provide the trial registration number **OR** cite DOI.	Not Applicable	
Report the **clinical trial registration number** (at ClinicalTrials.gov or equivalent), where applicable.	Not Applicable	

Laboratory protocol	Information included in the manuscript?	In which section is the information available? (Reagents and Tools Table, Materials and Methods, Figures, Data Availability Section)
Provide DOI OR other citation details if **external detailed step-by-step protocols** are available.	Not Applicable	

Experimental study design and statistics	Information included in the manuscript?	In which section is the information available? (Reagents and Tools Table, Materials and Methods, Figures, Data Availability Section)
Include a statement about **sample size** estimate even if no statistical methods were used.	Yes	Materials and Methods
Were any steps taken to minimize the effects of subjective bias when allocating animals/samples to treatment (e.g. **randomization procedure**)? If yes, have they been described?	Yes	Materials and Methods
Include a statement about **blinding** even if no blinding was done.	Yes	Materials and Methods
Describe **inclusion/exclusion criteria** if samples or animals were excluded from the analysis. Were the criteria pre-established?<br><br>If sample or data points were omitted from analysis, report if this was due to attrition or intentional exclusion and provide justification.	Yes	Materials and Methods
For every figure, are **statistical tests** justified as appropriate? Do the data meet the assumptions of the tests (e.g., normal distribution)? Describe any methods used to assess it. Is there an estimate of variation within each group of data? Is the variance similar between the groups that are being statistically compared?	Yes	Materials and Methods

Sample definition and in-laboratory replication	Information included in the manuscript?	In which section is the information available? (Reagents and Tools Table, Materials and Methods, Figures, Data Availability Section)
In the figure legends: state number of times the experiment was **replicated** in laboratory.	Yes	Figure legends
In the figure legends: define whether data describe **technical or biological replicates**.	Yes	Figure legends

Ethics

Ethics	Information included in the manuscript?	In which section is the information available? (Reagents and Tools Table, Materials and Methods, Figures, Data Availability Section)
Studies involving **human participants**: State details of **authority granting ethics approval** (IRB or equivalent committee(s), provide reference number for approval.	Not Applicable	
Studies involving **human participants**: Include a statement confirming that **informed consent** was obtained from all subjects and that the experiments conformed to the principles set out in the WMA Declaration of Helsinki and the Department of Health and Human Services Belmont Report.	Not Applicable	
Studies involving **human participants:** For publication of **patient photos**, include a statement confirming that consent to publish was obtained.	Not Applicable	
Studies involving experimental **animals**: State details of **authority granting ethics approva**l (IRB or equivalent committee(s), provide reference number for approval. Include a statement of compliance with ethical regulations.	Yes	Materials and Methods
Studies involving **specimen and field samples:** State if relevant **permits** obtained, provide details of authority approving study; if none were required, explain why.	Not Applicable	

Dual Use Research of Concern (DURC)	Information included in the manuscript?	In which section is the information available? (Reagents and Tools Table, Materials and Methods, Figures, Data Availability Section)
Could your study fall under dual use research restrictions? Please check biosecurity documents and list of **select agents and toxins** (CDC): https://www.selectagents.gov/sat/list.htm	Not Applicable	
If you used a select agent, is the security level of the lab appropriate and reported in the manuscript?	Not Applicable	
If a study is subject to dual use research of concern regulations, is the name of the **authority granting approval and reference number** for the regulatory approval provided in the manuscript?	Not Applicable	

Reporting

The MDAR framework recommends adoption of discipline-specific guidelines, established and endorsed through community initiatives. Journals have their own policy about requiring specific guidelines and recommendations to complement MDAR.

Adherence to community standards	Information included in the manuscript?	In which section is the information available? (Reagents and Tools Table, Materials and Methods, Figures, Data Availability Section)
State if relevant guidelines or checklists (e.g., **ICMJE, MIBBI, ARRIVE, PRISMA**) have been followed or provided.	Not Applicable	
For **tumor marker prognostic studies,** we recommend that you follow the **REMARK** reporting guidelines (see link list at top right). See author guidelines, under 'Reporting Guidelines'. Please confirm you have followed these guidelines.	Not Applicable	
For **phase II and III randomized controlled trials**, please refer to the **CONSORT** flow diagram (see link list at top right) and submit the CONSORT checklist (see link list at top right) with your submission. See author guidelines, under 'Reporting Guidelines'. Please confirm you have submitted this list.	Not Applicable	

Data Availability

Data availability	Information included in the manuscript?	In which section is the information available? (Reagents and Tools Table, Materials and Methods, Figures, Data Availability Section)
Have **primary datasets** been deposited according to the journal's guidelines (see 'Data Deposition' section) and the respective accession numbers provided in the Data Availability Section?	Yes	Data Availability
Were **human clinical and genomic datasets** deposited in a public access-controlled repository in accordance to ethical obligations to the patients and to the applicable consent agreement?	Not Applicable	
Are **computational models** that are central and integral to a study available without restrictions in a machine-readable form? Were the relevant accession numbers or links provided?	Not Applicable	
If publicly available data were reused, provide the respective **data citations in the reference list**.	Not Applicable	
